# The *LATERAL ORGAN BOUNDARIES Domain* gene family in grapevine: genome-wide characterization and expression analyses during developmental processes and stress responses

**DOI:** 10.1038/s41598-017-16240-5

**Published:** 2017-11-21

**Authors:** Jérôme Grimplet, Diana Pimentel, Patricia Agudelo-Romero, Jose Miguel Martinez-Zapater, Ana Margarida Fortes

**Affiliations:** 1 Instituto de Ciencias de la Vid y del Vino (CSIC-Universidad de La Rioja-Gobierno de La Rioja), 26006 Logroño, Spain; 2 Universidade de Lisboa, Faculdade de Ciências de Lisboa, BioISI, Campo Grande, 1749-016 Lisboa Portugal; 3 Present Address: The UWA Institute of Agriculture, The University of Western Australia, M082 Perth, 6009, Australia and the ARC Centre of Excellence in Plant Energy Biology, The University of Western Australia, M316 Perth, Perth, 6009 Australia

## Abstract

LATERAL ORGAN BOUNDARIES (LOB) DOMAIN (LBD) constitute a family of plant-specific transcription factors with key roles in the regulation of plant organ development, pollen development, plant regeneration, pathogen response, and anthocyanin and nitrogen metabolisms. However, the role of LBDs in fruit ripening and in grapevine (*Vitis vinifera* L.) development and stress responses is poorly documented. By performing a model curation of *LBDs* in the latest genome annotation 50 genes were identified. Phylogenetic analysis showed that *LBD* genes can be grouped into two classes mapping on 16 out of the 19 *V. vinifera* chromosomes. New gene subclasses were identified that have not been characterized in other species. Segmental and tandem duplications contributed significantly to the expansion and evolution of the *LBD* gene family in grapevine as noticed for other species. The analysis of *cis*-regulatory elements and transcription factor binding sites in the *VviLBD* promoter regions suggests the involvement of several hormones in the regulation of *LBDs* expression. Expression profiling suggest the involvement of LBD transcription factors in grapevine development, berry ripening and stress responses. Altogether this study provides valuable information and robust candidate genes for future functional analysis aiming to clarify mechanisms responsible for the onset of fruit ripening and fruit defense strategies.

## Introduction

Transcription factors play an important role in the regulation of plant development and disease response. Among them, LATERAL ORGAN BOUNDARIES DOMAIN (LBD) proteins defined by a conserved N-terminal LATERAL ORGAN BOUNDARIES (LOB) domain is a family of plant-specific transcription factors with key roles in the regulation of plant organ development^1,2^. The heterodimeric interactions between the Arabidopsis AS1, AS2, and JLO proteins are involved in the establishment of organ boundaries^3^. AS2 (LBD6) interacts with AS1 in the process of leaf formation and are known to be required for repression of meristematic genes and establishment of leaf adaxial-abaxial polarity^4^. These proteins are also involved in the development of sepal and petal primordia of flowers by repressing boundary-specifying genes for normal development of the organ^5^. JLO/LBD30 is a general regulator of cell specification and organ patterning throughout plant development^6^. On the other hand, LBD16, LBD18, and LBD29 regulate lateral root organogenesis in Arabidopsis as direct targets of Aux/IAA–ARF modules in the auxin signalling pathway^7^.

Recent studies showed that these proteins are also involved in pollen development, plant regeneration, photomorphogenesis, pathogen response, and anthocyanin and nitrogen metabolisms^2^. In this way, LBD27/SIDECAR POLLEN (SCP) and LBD10 are Arabidopsis microspore-specific LBD proteins having cooperative and unique roles in male gametophyte development^8^. The Arabidopsis LBD proteins LBD16, LBD17, LBD18, and LBD29 are key regulators in the callus induction process associated with plant regeneration, and establish a molecular link between auxin signalling and the plant regeneration program^9^. Arabidopsis *LBD*2*0* is a *Fusarium oxysporum* susceptibility gene that appears to regulate components of jasmonic acid (JA) signalling required for full elicitation of *F. oxysporum*- and JA-dependent responses^10^. Arabidopsis LBD25/DDA1 is involved in the regulation of light/dark-dependent hypocotyl elongation^11^. LBD proteins have also been involved in developmental processes in non-model plants such as secondary phloem growth in Populus^12^ and pulvinus differentiation and petiole development in legumes^13^.

The characteristic LOB domain comprises a C-block containing four cysteine with spacing (CX2CX6CX3C) required for DNA-binding activity, a Gly-Ala-Ser (GAS) block and a leucine zipper- like coiled-coil motif (LX6LX3LX6L) responsible for protein dimerization^1,2^. Several LBD proteins are capable to form homo- and hetero- dimers^2,3,8,14^. Recently, it was demonstrated that the conserved proline residue in the GAS block is also crucial for the DNA-binding activity of Arabidopsis LBD16 and LBD18 proteins which have a role in lateral root formation^15^. The LBD gene family can be divided into two classes according to the structure of the LOB domain^16,17^. Class I *LBD* genes contain a perfectly conserved CX2CX6CX3C zinc finger-like domain and an LX6LX3LX6L leucine zipper-like coiled-coil motif, whereas class II *LBD* genes only have a conserved zinc finger-like domain^17^. The majority of *LBD* genes belong to class I. Class II LBD proteins have an incomplete, probably not functional, leucine zipper that cannot form a coiled–coil structure^1^.

Functional analysis, mainly in Arabidopsis, rice and maize, revealed that class I *LBD* genes are mostly involved in plant development such as lateral organ (leaf and flower) development^1,2^, and in auxin signal transduction cascade that leads to the formation of lateral roots^7,18–20^. By contrast, class II genes seem to be involved in metabolism, particularly as repressors of anthocyanin synthesis and N availability signals in the plant^21,22^.

LOB domain proteins are suggested to act as transcription factors based on their nuclear localization^7,23^, and their capacity to bind to DNA motifs^24^. The DNA-binding affinity of ASL4 (LOB) was reduced by interacting with bHLH048 proteins^24^. The variable C-terminal region of LBD proteins confers transcriptional control on downstream gene expression^14^.

*In silico* genome analyses predicted the presence of 43 *LBD* members in *Arabidopsis thaliana* and *Zea mays*, 35 in *Oryza sativum* and 58 in *Malus domestica* ^17,25–27^. As more species have their complete reference genome sequenced, additional *LBD* genes can be identified and the biological roles of this poorly studied gene family clarified.

Grapevine has been a widely-studied species during the last decade at the genomics level. The release of the whole grapevine genome sequence in 2007 represented a breakthrough to promote its molecular genetics analysis^28^. Based on the published sequence data, comprehensive analysis of a given gene family can be performed to uncover its molecular functions, evolution and gene expression profiles. These analyses can contribute to the understanding of how genes in gene families control traits at a genome-wide level.

Recent preliminary analyses predicted 40 *LBD* genes in the grapevine genome^29^ using an older version of the grapevine genome and without manual curation. In this work, we have identified 50 LBD genes and have performed a detailed structural analysis and mapping of these genes on the grapevine chromosomes. This gene family has been compared with similar families in thirty-three plant species. Finally, identification of c*is*-acting regulatory elements in promoter regions together with expression analyses based on microarray and RNAseq data suggest that *LBD* proteins are involved in the process of grape ripening and in the plant response to abiotic and biotic stresses.

## Results

### Structural annotation of LBD genes, phylogenetic analysis, and nomenclature

Genes that were previously identified as *LATERAL ORGAN BOUNDARIES DOMAIN* in the grapevine genome^30^ were used to performed sequence comparison analyses with BLASTX, either against the most up to date gene predictions from CRIBI V1 and V2, the NCBI refseq (remapped on the 12Xv2 of the genome assembly) and the VCOST (on the 12Xv2 of the genome assembly). Analyses were also performed directly against the reference genome sequence with TBLASTX to check whether any potential gene could have been missed by these predictions. By using these approaches, we identified 50 genome regions that shared homology with at least one of the genes.

Gene models were curated using the data collected from gene structure comparisons using different databases as well as the available inflorescence and flower RNAseq data from the laboratory (data not shown). RNAseq data allowed to evaluate whether newly detected genes, not represented in microarray data, showed expression, by redoing the bioinformatics analysis of original RNAseq data with an updated GFF file. A total of 50 *LBD* genes having a putatively functional structure were identified in the grapevine genome (Table 1), which is similar to the number of genes identified in Arabidopsis genome (43 genes)^16,17^. Data relative to the detection of *LBD* genes in previous genome annotations or gene-sets are summarized in Supplementary Table S1. The majority of the genes were identified in all the annotations. However, four genes were not detected in the automatic annotation CRIBIv1, three were not detected in the CRIBIv2, six were missing in the VIB annotation, and two in the NCBI refseq annotation. Representative sequences for each gene model were selected from the different annotations based on their quality (apparently full length gene when compared to other species, no chimera): 13 were selected from the CRIBI, 2 from the VIB annotation and the remaining 35 from the refseq annotation. These genes are integrated in the Grapevine annotation V3 recently published^31^.

**Table 1:** Genome localization of the 50 grapevine *VviLBD* genes.

Locus ID	Short Name	Strand	Position	Locus ID	Short Name	Strand	Position
Vitvi08g00144	LBDIa1 LBD20	+	2648151–2649186	Vitvi13g00551	LBDIf5	−	5061981–5063306
Vitvi15g00736	LBDIa2 LBD19	+	14992229–14994929	Vitvi13g00552	LBDIf6	−	5073630–5074839
Vitvi15g00735	LBDIa3	−	14983081–14985200	Vitvi13g00549	LBDIf7	+	5039284–5040392
Vitvi07g00573	LBDIa4 LBD16	−	6228524–6229895	Vitvi13g00545	LBDIf8	+	4985620–4986382
Vitvi13g00333	LBDIa5 LBD33	+	3457062–3457909	Vitvi13g00546	LBDIf9	+	5011144–5011863
Vitvi07g00572	LBDIa6	+	6220003–6220810	Vitvi13g00556	LBDIf10	−	5144789–5155693
Vitvi17g00890	LBDIc1 LOB	−	10710469–10712731	Vitvi13g00543	LBDIf11	+	4954628–4956343
Vitvi14g01707	LBDIc2	+	27155871–27157919	Vitvi06g00336	LBDIf12	−	4180846–4182157
Vitvi13g00085	LBDIc3 LBD21	−	817035–817745	Vitvi06g00338	LBDIf13	−	4201430–4202390
Vitvi00g00480	LBDIc4 LBD6	+	11326893–11330106	Vitvi16g01446	LBDIg1	+	17405415–17406086
Vitvi00g01060	LBDIc5	−	22493178–22495116	Vitvi15g01216	LBDIi1	+	17253530–17254344
Vitvi07g01328	LBDIc6	−	18720228–18722222	Vitvi15g01217	LBDIi2	+	17259993–17261012
Vitvi07g01326	LBDIc7	−	18699491–18702938	Vitvi04g01768	LBDIi3	+	996279–997019
Vitvi07g01327	LBDIc8	−	18708083–18709964	Vitvi14g01878	LBDIi4 LBD27	−	28646091–28646927
Vitvi16g00859	LBDIc9	−	15931002–15932161	Vitvi17g00520	LBDIi5	−	6117662–6119015
Vitvi19g01589	LBDId1 LBD3	+	21536622–21539654	Vitvi09g00188	LBDIi6 LBD22	−	2066342–2067873
Vitvi10g01237	LBDId2 LBD4	−	17047348–17048794	Vitvi12g00230	LBDIi7 LBD2	−	3392281–3394534
Vitvi06g00706	LBDId3	+	7971884–7972497	Vitvi11g00169	LBDIi8	+	1720665–1722595
Vitvi13g00109	LBDId4	+	1022072–1022675	Vitvi14g01193	LBDIIa1	+	21211055–21212346
Vitvi13g00144	LBDId5	−	1309999–1311255	Vitvi17g00325	LBDIIa2	−	3791838–3793171
Vitvi06g00772	LBDId6 LBD13	−	8584220–8586134	Vitvi01g00291	LBDIIa3	−	3210258–3211492
Vitvi13g01866	LBDIf1	−	5100511–5101131	Vitvi01g00290	LBDIIa4	+	3204504–3205665
Vitvi13g01867	LBDIf2	−	5102158–5103523	Vitvi18g00677	LBDIIb1	+	7746353–7747276
Vitvi13g00555	LBDIf3	−	5130143–5136580	Vitvi07g01610	LBDIIb2 LBD39	+	21897655–21899042
Vitvi13g00559	LBDIf4	−	5173575–5179644	Vitvi03g00628	LBDIIc1	−	7098961–7099834

**Figure 1:**
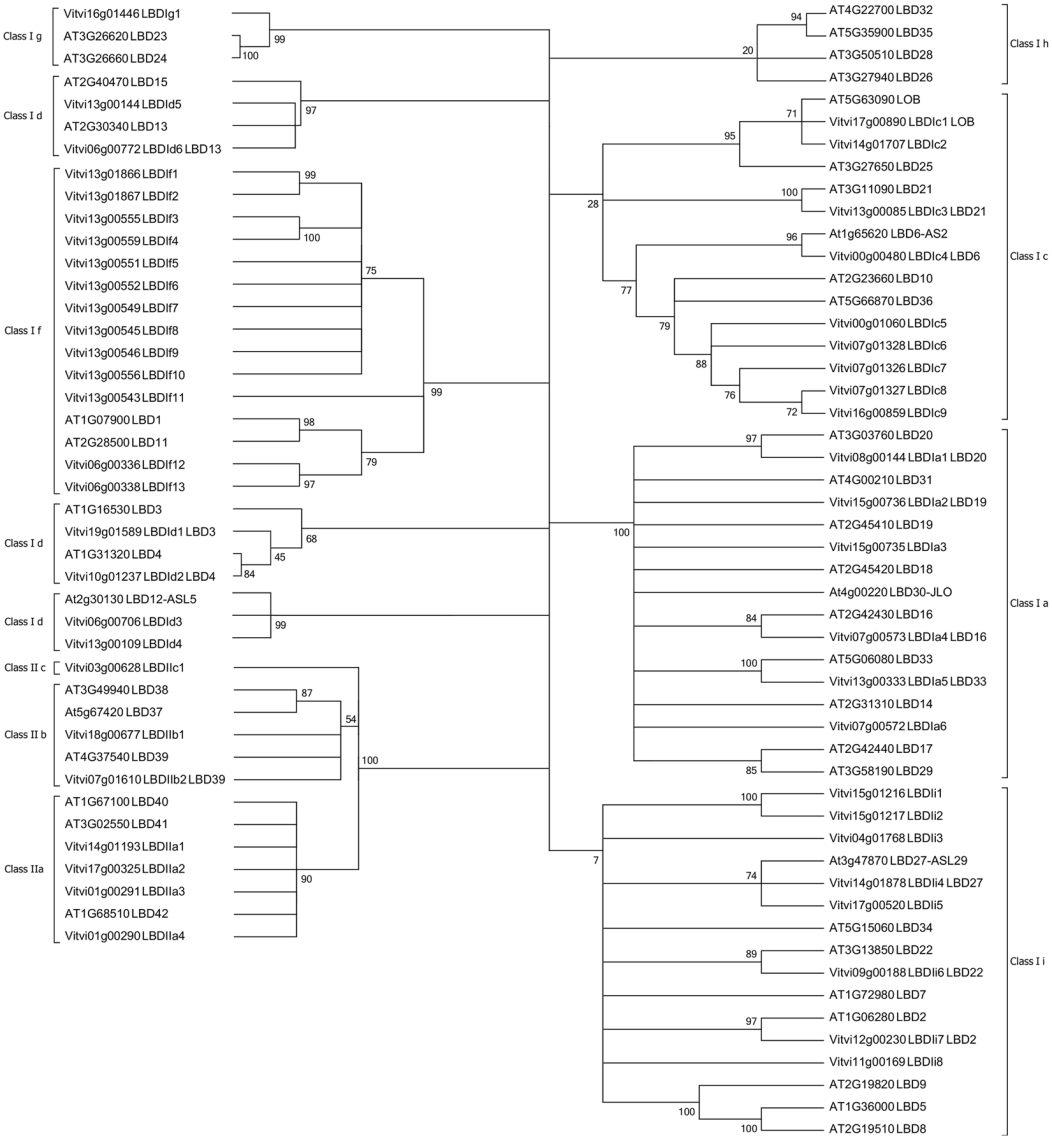
Phylogenetic analysis of grapevine and Arabidopsis *LBD* genes. Two classes were identified, Class I subdivided into six subclasses (**a**,**c**,**d**,**f**,**g**, and **i**) and Class II into three (**a**–**c**).

Regarding nomenclature, a phylogenetic tree of the LBD protein coding genes in *V. vinifera* and Arabidopsis was constructed (Fig. 1) as suggested by the Super-Nomenclature Committee for Grape Gene Annotation (sNCGGa)^32^. A bootstrap value of 70 as recommended by the Committee allowed to discriminate the genes within the majority of the classes but for some of them the phylogenetic analysis was complemented by motif analysis to detect conservation within classes and determine the affiliation of the genes inside some classes. The use of lower bootstrap values allowed to retrieve the same classes as in Arabidopsis. Class d is the only family where the genes are not all within the same branch. The genes are part of a subtree with the class f, but all the genes clustered with an Arabidopsis gene from class Id with a bootstrap value higher than 70. Class Ic is hardly conserved with a bootstrap of 28 necessary to maintain the tree architecture. However, clear consensus is found in the GAS motif and all the genes clustered with an Arabidopsis gene from class Ic with a bootstrap value higher than 70. Class IIb require a bootstrap of 54 to maintain the tree architecture but also clear conservation is observed in the LX6LX3LX6 motif. Class Ii requires a bootstrap of 7 to maintain the tree architecture, which is rather low and four genes were not clustering with an Arabidopsis gene from class Ic with a bootstrap value higher than 70. As in other species, *VviLBD* genes fall into two classes: Class I with 43 genes and Class II with 7 genes, relative to 37 and six in Arabidopsis^16,17^. Class I *VviLBD* genes were grouped into six subclasses (a, c, d, f, g, and i) and Class II genes into three subclasses (a–c). Arabidopsis *LBD* genes were not clustered in subclass IIc, which includes only the *VviLBDIIc1* gene. Only two Arabidopsis *LBD* genes (*LBD1* and *LBD11*) were grouped in subclass If with thirteen *VviLBDIf (1–13)* genes. For individual gene nomenclature, since both Arabidopsis nomenclature and previously named *Vitis* genes were named based on a poorly informative numeric code and few clear orthologs were identified, gene symbols/names were adapted to the class, the subclass and a distinctive number as proposed for *Vitis* genes nomenclature^32^. Correspondences among different nomenclatures are described in Supplementary Table S1.

Regarding the exon/intron structure (Supplementary Fig. S1), the majority of the *VviLBD* genes presented two exons (37 genes), as it is commonly observed in other plant species^25,26,33,34^. Nevertheless, four of them have a non-coding exon (*LBDIc1 LOB*, *LBDIc6*, *LBDIc8*, *LBDIi1*), while *LBDIc6* expression was not detected according to RNAseq data. Thirteen genes present a different exon/intron structure comparing to the other 37 genes: five of them did not have any intron (*LBDIc2*, *LBDIc3,* *LBDIc9*, *LBDIi3*, and *LBDIi4)*, and seven of them contained three exons (*LBDIc5*, *LBDId5*, *LBDId6,* *LBDIf3*, *LBDIf4*, *LBDIi7,* and *LBDIi8*). However, *LBDIc5* presented two non-sense exons. Finally, *LBDIc4* presented five exons, although four of them were predicted as non-sense. Four of the five genes with predicted non-sense exons belong to Class Ic. The size of the *LBD* gene locus varied ten times, ranging from 603 nucleotides (*VviLBDId4*) to 6437 nucleotides (*VviLBDIf3*).

### Motif analyses and orthologous relationships

**Figure 2:**
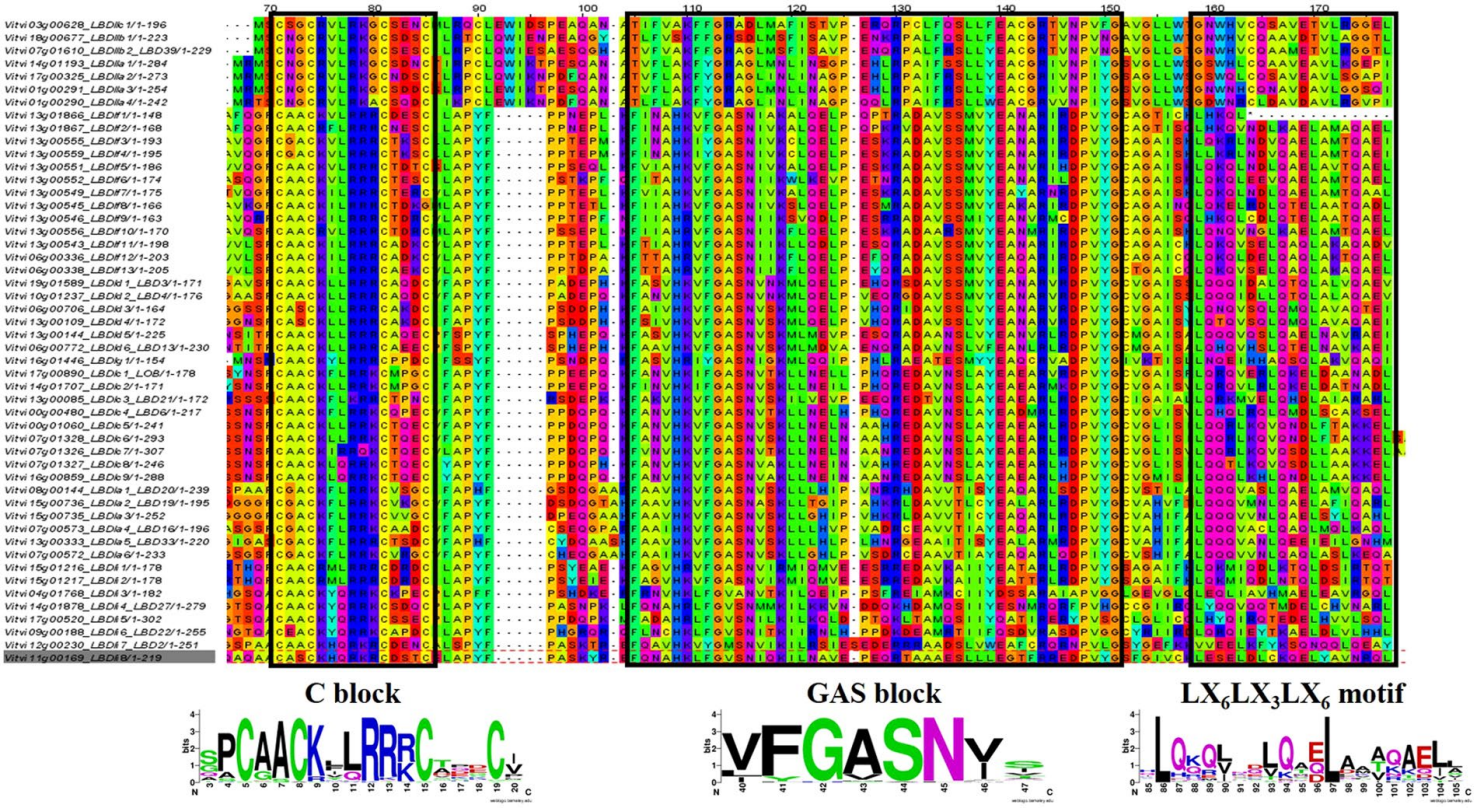
VviLBD protein alignment and motif analysis. Conserved domains were highlighted with black boxes. CX_2_CX_6_CX_3_C zinc finger-like domain was conserved in all 50 predicted VviLBD protein sequences while the leucine zipper-like motif (LX_6_LX_3_LX_6_L) was observed only in the class I VviLBD proteins. Details on protein structure are shown in Supplementary Fig. S1.

The LBD transcription factor family has a conserved LOB domain in the N terminus that comprises a C-block, a GAS block and a leucine-zipper-like coiled-coil motif^16,17^. Multiple sequence alignment within all of the VviLBD predicted proteins showed that the CX_2_CX_6_CX_3_C zinc finger-like domain was conserved in all 50 predicted protein sequences (Fig. 2, Supplementary Fig. S2). In addition, VviLBD proteins had a completely conserved G amino acid at the GAS block (Fig. 2). Class I LBD proteins presented a phenylalanine (F) and a histidine (H) completely conserved at the FX2(V/A)H motif, which represents the beginning of the GAS block. At the DP(V/I) YG motif of the Arabidopsis LBD proteins^17^, the proline (P) and the glycine (G) were completely conserved in all predicted grapevine proteins. The conserved proline residue in the GAS block was demonstrated in Arabidopsis to be essential in the biological function of the LBD proteins, since their replacement by leucine residues precludes LBD18-dependent control of the lateral root development via inhibition of the DNA-binding activity^15^. Valine (V) and leucine residues in the GAS block as well as a glutamine (Q) in the leucine zipper-like motif were found to be needed for motor organ specification in pea^13^. As observed for other plant species, leucine zipper-like motif (LX_6_LX_3_LX_6_L) was observed only in Class I VviLBD proteins and absent in Class II proteins, which suggests distinct functions of both classes. N and C terminals beyond the 3 blocks were not conserved at all among any sequences indicating that they probable play only a marginal role in protein function (Supplementary Fig. S2). It is however noteworthy that none of class II proteins presents a N terminal but this is not specific of the class; other class I proteins do not have it either. Class If protein present a longer, serine-enriched N terminal.

**Figure 3:**
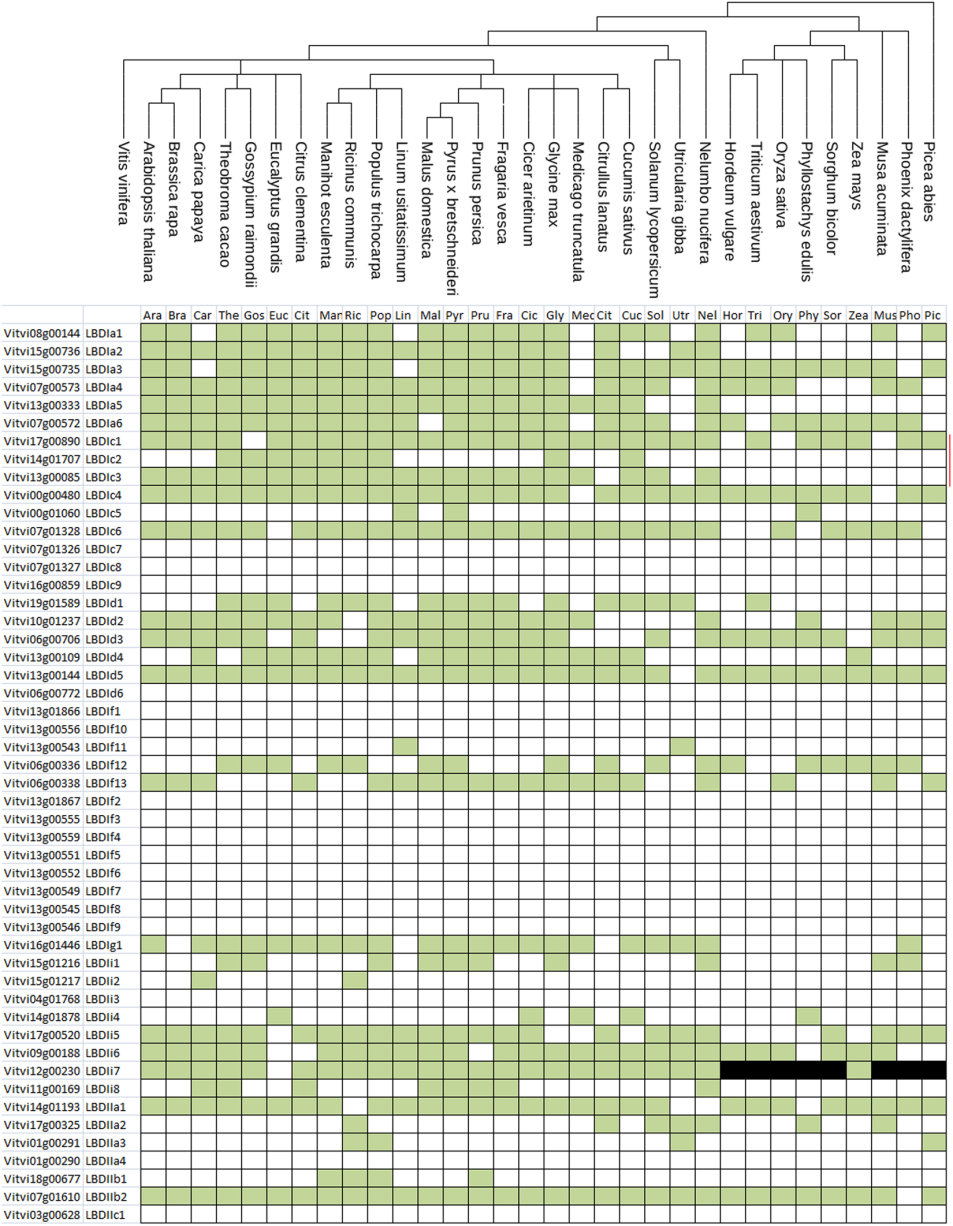
Grapevine LBD genes orthology against plant species with sequenced genomes. Green color represents one-to-one orthologs in the species (ortholog one-to-one = best match in the species that has the grapevine deduced protein as the best match in grapevine); white color represents no one-to-one homology match, and black color represents no match in the species.

The orthologous relationship of *LBD* genes in *V. vinifera* and other plant species was analysed as previously described^35^ (Fig. 3). Orthologous relationships were classified into two categories depending on whether or not a one-to-one relationship with a given species gene was detected. Since the 3 blocks previously mentioned were highly conserved, homology between a grapevine gene and many LBD genes was systematically detected, except for *VviLBDIi7* with most monocot species (in black in Fig. 3). Twenty genes showed a one-to-one orthologue relationship with an Arabidopsis gene when the comparison was carried out only with Arabidopsis. These genes likely correspond to well-conserved functions between both species.

In this context, a phylogenetic tree considering several mono and dicotyledonous species was constructed to identify genes with widely conserved functions among species (Fig. 3, Supplementary Fig. S3). *VviLBDIa3, VviLBDIc1, VviLBDIc4, VviLBDId5*, *VviLBDIIa1* and *VviLBDIIb2* presented orthologues at least in 88% of the species selected for comparison and could be involved in evolutionarily conserved functions.

This analysis did not detect orthologs for seventeen *LBD* genes while less than five orthologs were detected for six genes, mainly belonging to subclass If and Class II. This results may indicate that those proteins play a specific role in grapevine and in fact, Class IIc seems to be a *Vitis vinifera* species-specific subgroup. Regarding *VviLBDIa2, VviLBDIa6* and *VviLBDc3* they might have evolved after the monocot-dicot divergence since no orthologs were identified for them in the analysed monocot species. Supplementary Fig. S3 shows a cluster of the *Vitis* genes from the LBD1f subclass indicating a possible duplication event that appeared later and might be specific of the *Vitis* genus. Additionally, a Ka/Ks analysis was performed using the Ka/Ks calculation tool (http://services.cbu.uib.no/tools/kaks) on all the orthologs detected in the species for each grapevine gene, but no positive selection involving a grapevine gene in our gene set could be detected (no branch showed Ka/Ks ≫ 1, Supplementary Table S1).

### Chromosomal location of the LBD genes

**Figure 4:**
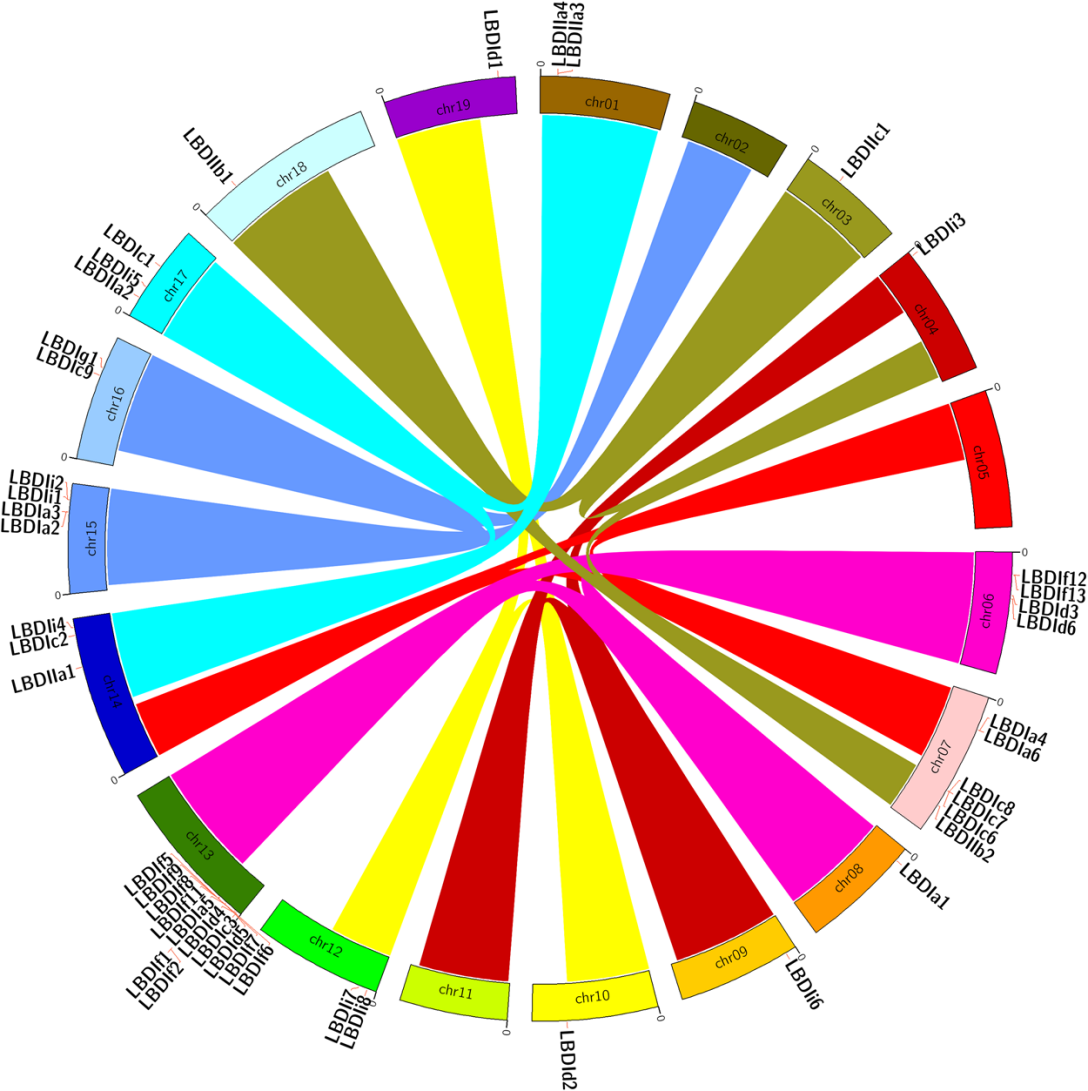
Chromosomal localization of grapevine *LBD* genes. Links with the same colors in different chromosomes show previously described paralogous regions^23^. *LBD* genes from the same subclass were located in chromosomal regions that were previously identified as paralogous segments.

Grapevine *LBD* genes are unevenly distributed among the nineteen chromosomes. They are located in all chromosomes, except on chromosomes 2, 5 and 11 (Fig. 4). Two genes, *LBDIc4 LBD6* and *LBDIc5*, were located on two scaffolds not assembled yet into any chromosome (they appear in the fictional chromosome “Unknown”). The highest number of *VviLBD* genes (15) was located on chromosome 13. The high number of *LBD* genes in this chromosome is mainly due to tandem repetition of genes belonging to the same subclass, in particular subclass *LBD* If genes. As highlighted by the orthology analysis, this duplication of class f probably occurred recently in *Vitis* since no ortholog was found in any other species. In contrast, chromosomes 3, 4, 8, 9, 10, 11, 12, 18, and 19 all carried a single *LBD* gene.

*LBD* genes belonging to the same subclass were located in chromosomal regions that were previously identified as paralogous segments resulting from ancestral polyploidization events^28,36^. In this way, *LBD* genes from subclass If are located in chromosomes 13 and 6, though LBD1f in chr 13 was located mostly just beside the presumed paralogous segment (Fig. 4). This is highly similar to what was obtained in a previous study for the GRAS sub-family LISCL^35^. The LISCL genes are also duplicated in the same area close to the paralogous region and have paralogs in Chr6. LBD1f and LISCL are at the same distance in chr13 and chr06 (1.7 Mb). It is possible that this area belongs actually to the paralogous region, since the paralog analysis was performed in the very original 8X version of the genome^28^ and might need an update. Class II genes are specific of two groups of paralogous segments, one group on chromosomes 1, 14, and 17 and another group on chromosomes 3, 7, 18. This indicates that all these subclasses predate the ancestral polyploidization events and likely play specific roles in grapevine since their functions were not redundant and were not discarded throughout evolution.

In addition, there are also tandem repetitions of genes belonging to different subclasses, like *VviLBDIa5, VviLBDId4-5* and *VviLBDIc3*. These data revealed that segmental duplication and tandem duplications contributed significantly to the expansion and evolution of the *LBD* gene family.

### *Cis*-acting regulatory elements in promoter regions

**Figure 5:**
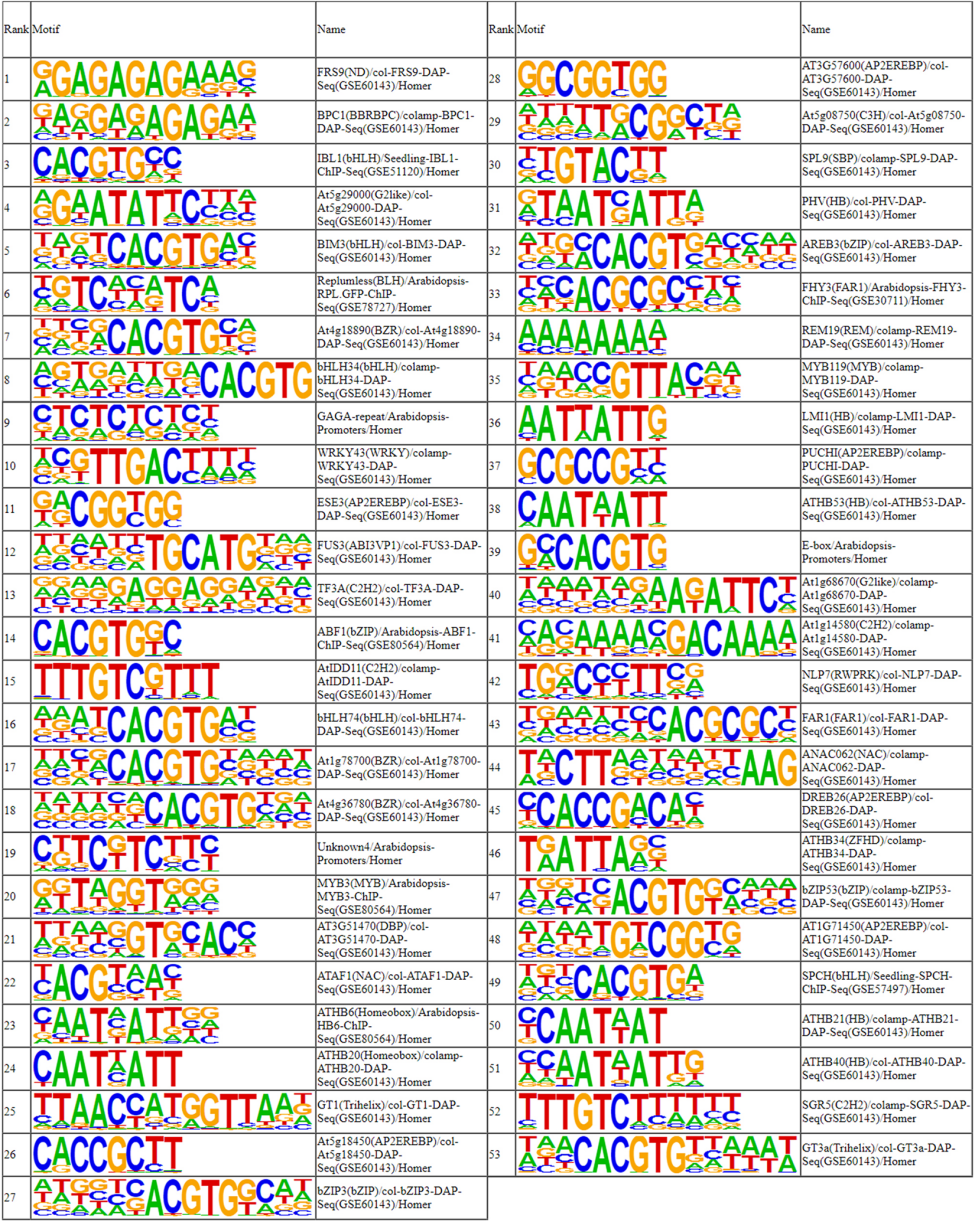
Enrichment of motifs on promoter regions of grapevine *LBD* genes. Several transcription factor binding sites were identified as enriched: FRS9 (ND); BPC1 (BBRBPC); IBL1 (bHLH); At5g29000 (G2like); BIM3 (bHLH); Replumless (BLH); At4g18890 (BZR); bHLH34 (bHLH); GAGA-repeat; WRKY43 (WRKY); ESE3 (AP2EREBP); FUS3 (ABI3VP1); TF3A (C2H2); ABF1 (bZIP); AtIDD11 (C2H2); bHLH74 (bHLH); At1g78700 (BZR); At4g36780 (BZR); Unknown4; MYB3 (MYB); AT3G51470 (DBP); ATAF1 (NAC); ATHB6 (Homeobox); ATHB20 (Homeobox); GT1 (Trihelix); At5g18450 (AP2EREBP); bZIP3 (bZIP); AT3G57600 (AP2EREBP); At5g08750 (C3H); SPL9 (SBP); PHV (HB); AREB3 (bZIP); FHY3 (FAR1); REM19 (REM); MYB119 (MYB); LMI1 (HB); PUCHI (AP2EREBP); ATHB53 (HB); E-box; At1g68670 (G2like); At1g14580 (C2H2); NLP7 (RWPRK); FAR1 (FAR1); ANAC062 (NAC); DREB26 (AP2EREBP); ATHB34 (ZFHD); bZIP53 (bZIP); AT1G71450 (AP2EREBP); SPCH (bHLH); ATHB21 (HB); ATHB40 (HB); SGR5 (C2H2); GT3a (Trihelix).

Analysis of *cis*-regulatory elements in the *VviLBD* promoter regions was performed using the PlantCARE (Supplementary Fig. S4; Supplementary Table S2) and PlantPAN databases (Supplementary Fig. S5; Supplementary Table S3). In addition to the core *cis-*elements, including the TATA box and CAAT box motifs presented in all promoter regions (data not shown), several regulatory motifs were identified and are associated with light regulation (BOX I, BOX 4, ACE, MRE), low temperature and heat stress responses (LTR, HSE), defence and stress responses (e.g. TC-rich repeats), hormonal regulation such as salicylic acid (e.g TCA-element, CA-element), methyl jasmonate (e.g CGTCA-motif), ethylene (e.g ERE), auxin (AuxRR-core, TGA-element), abscisic acid (ABRE, motif IIb, CE3), gibberellin (P-box, TATC-box, GARE-motif) and regulatory motifs related to tissue-specific expression (e.g Skn-1_motif, motif I, as1, GCN4_motif, RY-element) or developmental processes/ cell differentiation (HD-Zip 1, HD-Zip 2). Several transcription factor binding sites were also identified which have been widely associated with developmental processes and with biotic and abiotic stress responses namely AP2/ERF, NAC, C_2_H_2_, SBP, WRKY, Myb, bZIP and bHLH binding sites. Furthermore, these *cis*-regulatory elements were enriched in *LBD* promoter regions (Fig. 5; at P value < 0. 01). Interestingly, analyses of *de novo* motifs using Homer platform enabled the identification of a new motif GGTTGAATACAC as being enriched in *VviLBD* promoters (Supplementary Table S4). A similar known motif to this one was identified as GATGGAATAC (Supplementary Table S4).

It should be highlighted that the enrichment in MYB binding sites is known to regulate the expression of genes involved in phenylpropanoid metabolism. Interestingly, *VviLBDIf6* co-expressed with a gene coding for MYB divaricate and *VviLBDIa1* with a gene coding for a bHLH family transcription factor (Table 2). The bHLH048 transcription factor was already found to interact with the AtASL4 from Arabidopsis, and its interaction reduces the affinity of *LOB* gene for its 6-bp GCGGCG consensus DNA motif^24^.

**Table 2:** Co-expression analysis of the *VviLBD* genes.

Unique_ID/Nimblegen probeset	Functional annotation	Functional Categories
**VIT_15s0046g00230**	**VviLBDIi1**	**LOB domain family transcription factor**
**VIT_15s0046g00240**	**VviLBDIi2**	**LOB domain family transcription factor**
**VIT_01s0011g03540**	**VviLBDIIa3**	**LOB domain family transcription factor**
VIT_10s0003g03490	GA 2-oxidase	Metabolism. Secondary metabolism. Terpenoid metabolism. Diterpenoid metabolism. Diterpenoid biosynthesis
**VIT_14s0006g02950**	**VviLBDIIa1**	**LOB domain family transcription factor**
VIT_12s0057g00170	Wound-induced	Response to stimulus. Stress response. Abiotic stress response. wounding
**VIT_07s0197g00040**	**VviLBDIc7**	**LOB domain family transcription factor**
VIT_07s0031g02270	Tonoplast monosaccharide transporter2	Transport overview. Electrochemical Potential-driven Transporters. Porters. Major Facilitator Superfamily. Sugar Porter
**VIT_14s0066g00680**	**VviLBDIc2**	**LOB domain family transcription factor**
VIT_11s0016g05450	Equilibrative nucleoside transporter ENT8 splice variant	Transport overview. Electrochemical Potential-driven Transporters. Porters. Equilibrative Nucleoside Transporter
**VIT_01s0011g03530**	**VviLBDIIa4**	**LOB domain family transcription factor**
VIT_08s0007g04480	Pectinesterase family	Cellular process.Cellular component organization and biogenesis.Cell wall organization and biogenesis.Cell wall metabolism.Cell wall modification.Pectin modification
**VIT_06s0004g07790**	**VviLBDId6 LBD13**	**LOB domain family transcription factor**
VIT_02s0025g02940	Caffeic acid O-3-methyltransferase	Metabolism. Secondary metabolism. Phenylpropanoid metabolism. Phenylpropanoid biosynthesis
VIT_12s0028g03580	Lectin-receptor like protein kinase 3	Signalling. Signalling pathway. Protein kinase
VIT_14s0068g01360	GEM-like protein 5	Cellular process. Cell growth and death
VIT_02s0025g02920	Quercetin 3-O-methyltransferase 1	Metabolism. Secondary metabolism. Phenylpropanoid metabolism. Flavonoid metabolism. Flavonoid biosynthesis
**VIT_15s0048g00830**	**VviLBDIa3**	**LOB domain family transcription factor**
VIT_18s0001g15390	Gaiacol peroxidase	Metabolism. Primary metabolism. Amino acid metabolism. Aromatic amino acid metabolism. Phenylalanine metabolism. Phenylalanine biosynthesis
VIT_17s0000g09030	Disease resistance protein (NBS-LRR class)	Diverse functions. Gene family with diverse functions. NBS-LRR superfamily
VIT_15s0048g00500	Pectinesterase family	Cellular process. Cellular component organization and biogenesis. Cell wall organization and biogenesis. Cell wall metabolism. Cell wall modification. Pectin modification
**VIT_13s0019g03780**	**VviLBDIf6**	**LOB domain family transcription factor**
VIT_07s0031g02280	MYB divaricata	Development. Reproductive development. Flower development
**VIT_08s0056g01650**	**VviLBDIa1 LBD20**	**LOB domain family transcription factor**
VIT_11s0103g00200	Anthranilate N-benzoyltransferase	Metabolism. Primary metabolism. Amino acid metabolism. Aromatic amino acid metabolism. Aromatic amino acid biosynthesis
VIT_01s0127g00860	Aborted microspores AMS	Regulation overview. Regulation of gene expression. Regulation of transcription. Transcription factor. bHLH family transcription factor
VIT_18s0001g15690	Endo-1,4-beta-glucanase	Cellular process. Cellular component organization and biogenesis. Cell wall organization and biogenesis. Cell wall metabolism. Cell wall catabolism. Cellulose catabolism
VIT_18s0001g15680	Cellulase	Cellular process. Cellular component organization and biogenesis. Cell wall organization and biogenesis. Cell wall metabolism. Cell wall catabolism. Cellulose catabolism
VIT_15s0021g02170	Chalcone and stilbene synthase	Metabolism. Secondary metabolism. Phenylpropanoid metabolism. Flavonoid metabolism. Flavonoid biosynthesis
**VIT_17s0000g05490**	**VviLBDIi5**	**LOB domain family transcription factor**
VIT_09s0002g04380	Plastidic glucose transporter 2	Transport overview. Electrochemical Potential-driven Transporters. Porters. Major Facilitator Superfamily. Sugar Porter
VIT_12s0059g02500	Constans-like 11	Development. Reproductive development. Flower development
VIT_18s0001g13580	Kinesin motor protein	Cellular process. Cellular component organization and biogenesis. Cytoskeleton organization and biogenesis. Microtubule organization and biogenesis. Microtubule-driven movement
VIT_03s0063g00510	Leucine-rich repeat	Diverse functions. Gene family with diverse functions. NBS-LRR superfamily
VIT_06s0009g01830	Invertase, neutral/alkaline	Metabolism. Primary metabolism. Carbohydrate metabolism. Monosaccharide metabolism. Galactose metabolism
VIT_07s0031g01870	Zinc finger (CCCH-type) family protein	Regulation overview. Regulation of gene expression. Regulation of transcription. Transcription factor. C3H family transcription factor
VIT_00s2422g00010	Hexokinase-2	Metabolism. Primary metabolism. Carbohydrate metabolism. Glycolysis Gluconeogenesis
VIT_00s0288g00050	V-type H+-transporting ATPase subunit G	Metabolism. Primary metabolism. Generation of metabolite precursors and energy. Electron transport. Respiratory-chain phosphorylation
VIT_19s0014g01240	Morphogenesis of root hair 1 MRH1	Development. Root development
VIT_18s0122g00910	Mlo5	Cellular process. Cell growth and death. Cell death
VIT_17s0000g07750	Zinc finger protein 5	Regulation overview. Regulation of gene expression. Regulation of transcription. Transcription factor. C2H2 family transcription factor
VIT_07s0005g01640	feronia receptor-like kinase	Signalling. Signalling pathway. Protein kinase
VIT_00s0225g00170	Peroxidase	Metabolism. Primary metabolism. Amino acid metabolism. Aromatic amino acid metabolism. Phenylalanine metabolism. Phenylalanine biosynthesis

For some genes the list of co-expression is not complete. Further details are presented in Supplementary Table 2.

### Expression analysis of grapevine *LBD* genes

**Figure 6:**
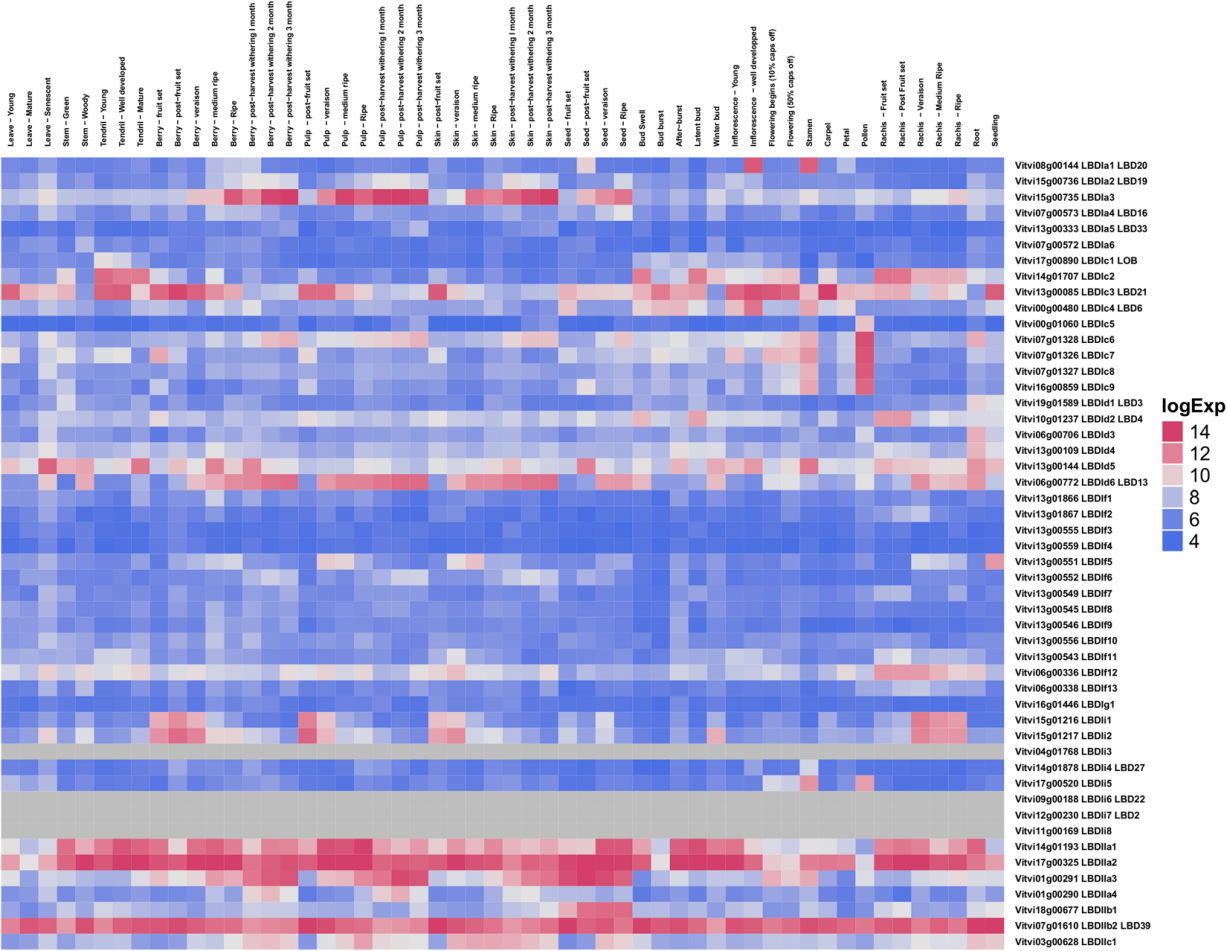
Expression of *LBD* genes in grapevine tissues. Gradient color is expressed in RMA-normalized intensity value on the Nimblegen microarray.

**Figure 7:**
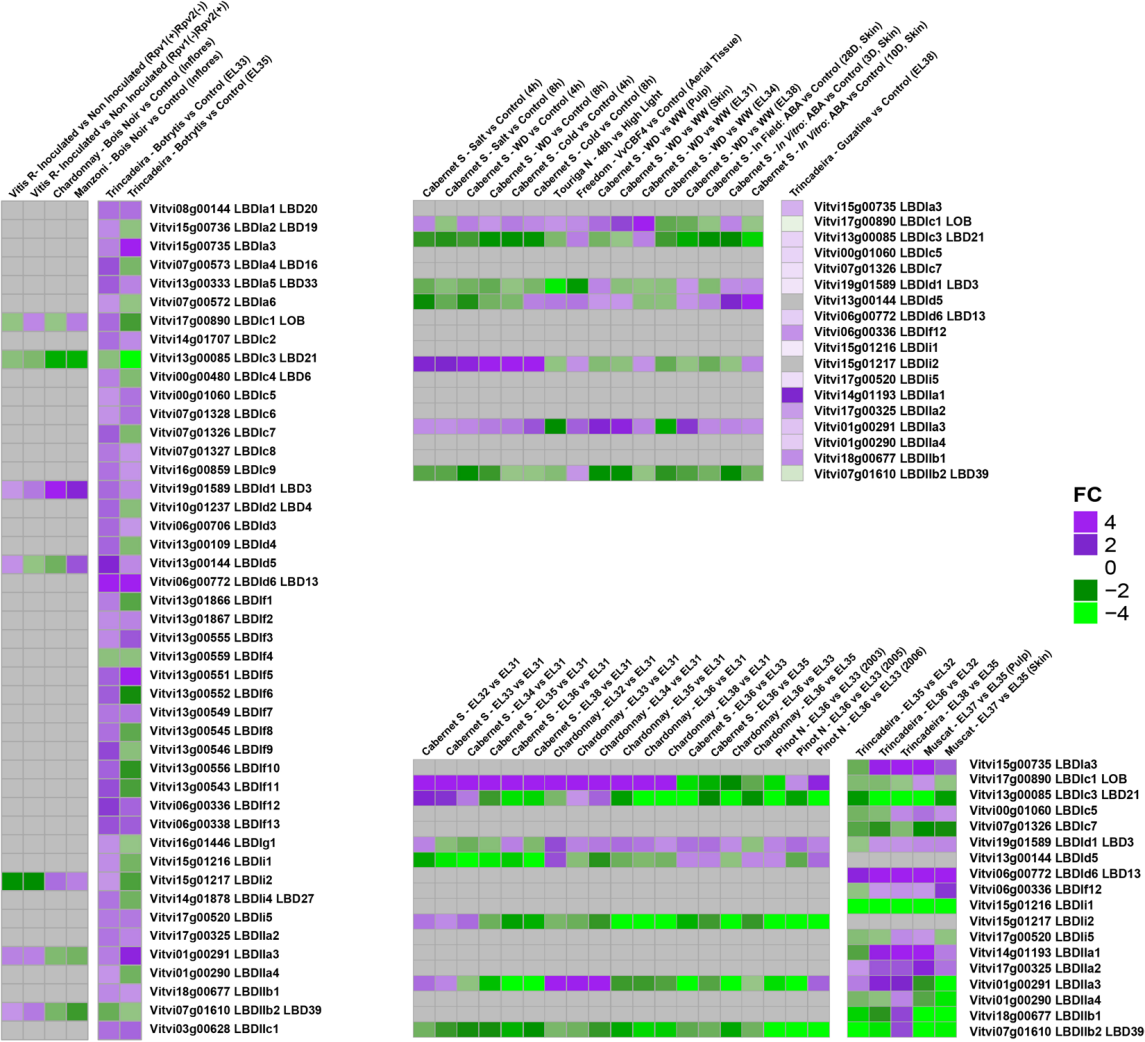
Expression of *LBD* genes during grape berry development and ripening, and upon abiotic and biotic stresses. Left experiments of each heatmap were performed with GeneChip microarrays, and right experiments were performed with GrapeGen microarray. Grape berry development: developmental stages from EL31 to EL38; cultivars Cabernet Sauvignon, Chardonnay, Pinot Noir, Trincadeira and Muscat. Abiotic stress experiments: salt, cold, water deficit, high light, ABA. Biotic stress experiments: *P. viticola*, *BoisNoir* and *Botrytis cinerea*.

Three distinct approaches were performed to characterize *LBD* genes expression in grapevine: (i) an atlas of expression of the *LBD* genes was constructed based on the absolute value of gene expression published in the grapevine gene expression atlas^37^ (Fig. 6). Plant Ontology (PO) was attributed when a gene was clearly expressed in a given tissue. (ii) co-expression analysis was preformed based on the same original data using relative values of expression of all genes, centered on the average expression (Supplementary Table S5). The main objectives of this analysis were to determine expression patterns and to identify genes that were following the same pattern as the *LBD* genes and that could be under the same regulatory elements, or under the regulation of the *LBD* gene itself. The results presented in Table 2 revealed that twelve genes showed a correlation with other genes with a Pearson Correlation Coefficient (PCC) threshold of 0.2. Finding the optimal PCC threshold to retrieve functionally related genes was affected by the method of gene expression database construction and the target gene function^38^, but the PCC that was chosen was very stringent. (iii) public expression data was analysed in order to identify the behaviour of *LBD* genes during berry development and ripening and upon abiotic and biotic stress conditions (Fig. 7). Figure 7 presented the expression value among the experiments where difference in expression of *LBD* genes was detected.

#### Tissue specific gene expression

Based on the *V. vinifera* cv. Corvina gene expression atlas^39^, several *LBD* genes showed a strong tissue specificity of expression, with the majority of Class I genes being poorly expressed in the different tissues (Fig. 6). *VviLBDIa3* and *VviLBDId6* were highly expressed mainly in ripe berry tissues. *VviLBDId6* was shown to be co-expressed (Table 2, Supplementary Table S5) with genes involved in phenylpropanoid metabolism, including two caffeic acid O-3-methyltransferase genes (VIT_02s0025g02940 and VIT_02s0025g02930) and a quercetin 3-O-metyltransferase gene (VIT_02s0025g02920), as well as with signaling and cell growth and death-related genes (VIT_12s0028g03580 and VIT_14s0068g01360, respectively). *VviLBDIc3* had high expression in young leaves, young and well developed tendril, inflorescences, and in berry tissues mainly at the beginning of fruit development (green and *véraison* stages). In addition, transcripts corresponding to subclass Ic genes, *VviLBDIc6-9*, seemed to be more abundant in pollen and stamen.

Interestingly, *VviLBDIa1* was only expressed in well-developed inflorescence and stamen and may have a specific function in the development of these tissues; it also co-expresses with genes involved in cell wall and secondary metabolism and transport (VIT_18s0001g15690, VIT_18s0001g15680, VIT_11s0103g00200, VIT_19s0015g00960). *VviLBDIc7* was co-expressed with *TONOPLAST MONOSACCHARIDE TRANSPORTER 2* (VIT_07s0031g02270). The class II genes *VviLBDIIa1, VviLBDIIa2*, and *VviLBDIIb2* were abundantly expressed in almost all grapevine tissues, and, *VviLBDIIa3* was more abundant in seeds and post-harvest berry tissues. Differential expression of *LBD* genes in diverse tissues were also observed for other plant species including Arabidopsis, rice, and maize^17,26,40,41^.

#### Gene expression during berry development and ripening

Expression studies regarding berry development and ripening revealed the involvement of *LBD* genes in different stages (Fig. 7). *VviLBDIc1* gene was highly expressed after EL31 stage^42^, i.e. pea-size berries, both in cv. Cabernet Sauvignon and cv. Chardonnay. However, this expression profile can be cultivar and/or season dependent. In cv. Trincadeira, *VviLBDId6* was expressed along berry ripening as previously mentioned for cv. Corvina. The same holds true for *VviLBDIa3* which showed up-regulation at the onset of ripening and was co-expressed with genes involved in stress response (Table 2, Supplementary Table S5) namely a Disease Resistance protein (NBS-LRR class) (VIT_17s0000g09030) and a Gaiacol peroxidase (VIT_18s0001g15390). On the other hand, *VviLBDIi1* was down-regulated in advanced ripening stages.

Concerning class II genes, *VviLBDIIa1, VviLBDIIa2, VviLBDIIa3* seem to play a role in grape ripening. Furthermore, *VviLBDIIa1* co-expressed with wound-induced genes involved in abiotic stress response and *VviLBDIIa3* co-expressed with a gene coding for GA 2-oxidase (VIT_10s0003g03490).

#### Gene expression upon abiotic stress

Expression analysis concerning abiotic stress conditions (Fig. 7) revealed that *VviLBDIi2* was up-regulated under salt, cold and water deficit conditions in shoot tips. *VviLBDId5* expression in berry skin showed a positive response to *in vitro* ABA treatment. *VviLBDIIa3* was also up-regulated in pulp and skin submitted to water deficit. Interestingly, *VviLBDIIa1* responded to guazatine treatment, an inhibitor of polyamine oxidase involved in polyamine catabolism^43^.

On the other hand, some *VviLBD* genes presented mostly down-regulation upon abiotic stress such as *VviLBDIc3* and *VviLBDIIb2. VviLBDId1* was strongly down-regulated after 48 h of high light exposure.

#### Gene expression upon biotic stress

Regarding biotic stress conditions (Fig. 7), *VviLBDIi2* was down-regulated in partially and completely resistant plants (resistance genes named Rpv1 and Rpv2) when inoculated with *Plasmopara viticola*. *VviLBDId1* was up-regulated in inflorescences presenting Bois noir disease, with higher expression in cv. Chardonnay. On the other hand, *VviLBDIc3* was down-regulated in the same conditions. This gene was also down-regulated in grape berries infected with *Botrytis cinerea*, with lower expression after long exposure (*véraison* stage). *VviLBDId6* and *VviLBDIIa1* were strongly up-regulated after *Botrytis* infection*. VviLBDIIa1* co-expressed with six wound-induced genes as previously mentioned (Table 2, Supplementary Table S5). *VviLBDIa3, VviLBDIf5* and *VviLBDIIa3* were up-regulated upon *Botrytis* infection with higher expression at *véraison* stage. However, the majority of *VviLBDs* seemed to participate in the early response towards *Botrytis* attack.

## Discussion

Prediction of putative biological functions for a given gene family can be approached based on genomic and transcriptomic available data with improved bioinformatics tools. In this study, we performed an extensive analysis of the *LBD* genes on the 12x *Vitis vinifera* genome sequence based on the isolation of the complete set of genes identified in PN40024. Characterization of *LBD* gene family and their putative functions was performed in grapevine based on detailed gene structure and expression analyses, chromosome localization, and comparative phylogenetic analyses with other sequenced genomes from different monocot and eudicot species.

### *LOB domain* gene family in grapevine

The *LOB domain* gene family, also known as *ASYMMETRIC LEAVES2*-like (*ASL*) gene family, was firstly described in the past decade^16,17^ and several studies have been made to unveil their role in plant processes. Specific LBD proteins characterized in Arabidopsis and in crop plants including rice seem to display a wide functional diversity. They were found involved in the regulation of several developmental processes, namely meristem programming, leaf patterning, inflorescence development, embryogenesis, lateral root formation, vascular patterning, as well as metabolic processes such as anthocyanin and nitrogen metabolisms^1,2^. More recently, they have been also associated with biotic stress responses^10,44–46^.

The exhaustive analysis of the grapevine *LBD* genes performed in this study has led to the identification of 50 *LBD* genes. The N-terminal LOB domain that characterizes this gene family was identified in all predicted proteins. The *VviLBD* genes were located to sixteen of the nineteen grapevine chromosomes. Phylogenetic analysis and evolutionary relationships divided the *LBD* gene family into two classes, Class I and Class II, as previously observed to other plant species, characterized by the presence or absence of functional leucine-zipper-like domains, respectively^16,17,25,26,33,40^. The majority of *VviLBD* genes belongs to Class I. Grapevine *LBD* genes were further clustered into nine subclasses, six in Class I and three in Class II. Minor clades of the two major classes were also identified in other plant species: class I was divided in five subgroups in rice and maize, four in Arabidopsis, *Lotus japonicus* and *Medicago truncatula*, and seven in *Malus domestica* ^25,26,40,47^. Class II was divided in two subclasses in apple and maize^25,40^.

The gene structure analysis revealed that 74% (37 out of 50) of the *VviLBD* genes contained two exons, as the majority of *LBD* genes in other plant species^25,26,47^, indicating a conserved structure during evolution. On the other hand, the highly variable C-terminal domain in LBD proteins from grapevine and other plant species indicate functional diversity associated with this gene family^47^. Experiments of LOB domain swapping for the *AS2* gene revealed that, despite the high similarity, the domain cannot be functionally replaced by a LOB domain of other family members indicating that dissimilar amino acid residues in the N-terminal are also important for the functional specificities of these transcription factors family members^47^. The highly-conserved C-block (CX_2_CX_6_CX_3_C) domain is present in all grapevine LBD proteins reinforcing its functional importance mainly associated with the DNA-binding process^17,47^. Class I proteins presented the leucine-zipper-like motif, which includes five hydrophobic amino acids (valine, isoleucine, leucine) separated by six variable amino acid residues and has been linked to protein-protein interaction.

The grapevine *LBD* gene family with 50 members is larger than the 43 *LBD* genes of Arabidopsis, the 24 in barley, the 31 in mulberry; the 35 in rice, 36 in *Sorghum bicolor*; the 38 in *Lotus japonicus*, and the 44 in maize^16,17,26,33,34,40,48–50^. *Medicago truncatula* and apple present a higher number of *LBD* genes, containing 57 and 58, respectively^25,33^. With approximately the same genome size, *Vitis vinifera* harbours more *LBD* genes than *Lotus japonicus* (approximately 487 and 470 Mb, respectively). Despite *Malus domestica* had almost the double genome size of *Vitis vinifera*, this fruit tree species only contains eight more genes^25^. Therefore, a large *LBD* density variation is observed among plant species.

Expansion of the grapevine *LBD* gene family likely took place by segmental/chromosomal duplication, as observed for other species from different taxonomic groups^25,34,47^. Duplicated genes like *VviLBDIi1 and VviLBDIi2* might show functional redundancy, as suggested by their similar expression profile or co-expression in several grapevine tissues. Their functional study might unveil the evolutionary role of gene duplication and their contribution in plant processes. Duplication events are more likely to be retained for gene families involved in signal transduction and transcriptional regulation^51^. Nevertheless, no additional grapevine LBD genes co-expressed together which might indicate different functions or specialization in most cases. In fact other likely duplicated genes such as *VviLBDIf12* and *VviLBDIf13*, showed a clear expression divergence in the expression analysis which might suggest their functional diversification. Tandem duplicated events were mainly associated with subclass If, which contained tandem repeated genes with high similarity. *VviLBDIf12* and *VviLBDIf13* presented several orthologs in other plant species, which might indicate conserved function. Nevertheless, for the remaining members of subclass If no ortholog was identified in the studied species, suggesting grapevine-specific functions. Moreover, some genes from specific subclasses were found in paralogous regions of the grapevine genome derived from polyploidization event^28^. Among them, subclass If had members in chromosomes 6 and 13, subclass IIa in chromosomes 1 and 17, and subclass IIb in chromosomes 7 and 18.

### Expression patterns across a variety of tissues indicate roles of LBD genes in regulation of metabolism and organ differentiation

*VviLBD* genes showed different expression patterns across the grapevine tissues. No subclass-specific expression pattern was observed, as occurred in other species namely *L. japonicus*, *M. truncatula* and apple, suggesting gene-specific function or localization regardless the phylogenetic subclass^25,33^. For example, *VviLBDIIb2* is highly expressed in almost all grapevine tissues whereas *VviLBDIIb1* seemed to be expressed only in seeds. *LBD38*, the Arabidopsis ortholog of *VviLBDIIb2*, is involved in nitrogen and anthocyanin metabolism, as well as their close homologs *LBD37* and *LBD39* ^21^. Moreover, the *LBD37* rice ortholog, *OsLBD37/ASL39*, was also associated with nitrogen metabolism, particularly in nitrogen remobilization and senescence^52^. This could suggest a conserved function of these genes across plant species and therefore *VviLBDIIb2* could regulate nitrogen and anthocyanin metabolism in a wide range of grapevines tissues. In fact, orthologs for this gene were found in 32 out of the 33 plants studied (Fig. 3).

Specific tissue expression patterns suggest the involvement of *VviLBDIa1* and *VviLBDIc3* in the development of floral organs. Interestingly, *VviLBDIa1* co-expressed with cell wall-related genes (endo-1,4-beta-glucanase, cellulose), secondary metabolism genes (anthranilate N-benzoyltransferase, chalcone and stilbene synthase) and with a bHLH family transcription factor (*aborted microspores AMS*). In rice, down-regulation of a LBD gene (*OsIG1*) led to developmental abnormalities of various floral organs^53^ providing links between LBD proteins and floral organ development as it could be the case in grapevine. Furthermore, in *Arabidopsis thaliana ASYMMETRIC LEAVES1* and *ASYMMETRIC LEAVES1 2* (*AS1* and *AS2*) and *JAGGED* (*JAG*) genes were shown to function in sepal and petal primordia to repress boundary-specifying genes for normal development of the organ^5^. In grapevine, the ortholog of the Arabidopsis *LBD6/AS2* is *VvLBDIc4* which is also expressed in flower organs but at a lower extent than *VviLBDIa1* and *VviLBDIc3*. Also noteworthy is the pollen specific expression of *VviLBDIc6*, *VviLBDIc7*, *VviLBDIc8* and *VviLBDIc9* which may have redundant functions. *VviLBDIc6* has orthologs in several species including *AtLBD36* which was previously shown to be expressed in pollen^54^.

### LBD genes may be involved in berry development and ripening through interaction with growth regulators

In grapevine, some *LBD* genes showed differential expression during fruit ripening, in particular the up-regulated genes *VviLBDIa3*, *VviLBDId6, VviBDIIa1* and the down-regulated *VviLBDIc3*, *VviLBDIi1* and *VviLBDIi2. VviLBDIIa3* was up-regulated at the initial development stages in cv. Chardonnay suggesting an involvement in fruit set and early developmental stages characterized by intense cell division. Nevertheless, *VviLBDIIa3* showed higher expression on the ripe stage of cv. Trincadeira, suggesting an expression pattern dependent on the variety. This gene is also co-expressed with a gene coding for gibberellin 2-oxidase (VIT_10s0003g03490) that inactivates endogenous bioactive gibberellins (GAs), suggesting an involvement of *VviLBDIIa3* in GA metabolism during fruit-set. In cv. Corvina, *VviLBDIIa3* was highly expressed in seed at fruit set and post-fruit set stages but also in ripe and post-harvest berries (Fig. 7). Little is known about the direct involvement of GAs on berry ripening, nevertheless, some evidences suggest a possible role in flowering and initial stages of berry development^55^. Additionally, differential accumulation of bioactive GAs was observed from flowering to fruit set, and this accumulation is finely regulated by the abundance and localization of GA oxidase transcripts^56^. Interestingly, *AtLBD40*, a close homolog of grapevine LBD subclass IIa genes, was reported to be a direct target of DELLA (growth-repressing transcription factor) in GA signalling pathway and to be down-regulated by gibberellin^57^.

*VviLBDId6*, expressed in ripe and post-harvest berry tissues, and also showed differential expression under biotic conditions. This gene was co-expressed with several genes involved in secondary metabolism (caffeic acid O-3-methyltransferase and quercetin 3-O-metyltransferase gene), signalling pathways (lectin-receptor like protein kinase 3) and cell growth and death (GEM-like protein 5). The expression of the close Arabidopsis homolog, *AtLBD15/ALS11* leads to down-regulation of several cellulose synthase genes and is activated by a key regulator of secondary cell wall synthesis^58^.

In *Vitis vinifera, LBD1d6* and *LBDIa3* were identified as positive molecular markers of ripening stage in three Portuguese cultivars^59^. Analysis of *cis*-acting elements suggests modulation of these genes by several growth regulators (ABA, methyl jasmonate, auxin, ethylene) and in response to stress (Supplementary Table S2). *VviLBDIa3* promoter showed a MYB binding site involved in flavonoid biosynthetic genes regulation. Interestingly, these two genes (VIT_06s0004g07790, VIT_15s0048g00830) were also identified as switch genes together with MYB transcription factors, cellulase, expansin B and caffeic acid 3-O-methyltransferase due to the fact that they are expressed at low levels in vegetative/green tissues and show a significant increase in mature/woody organs, suggesting a potential regulatory role during this developmental transition^60^. The putative participation of *LBD* genes in fruit ripening as suggested here is additionally supported by studies in banana where *Ma LBD 1 3* was found to be ripening inducible^61^.

Interestingly, the promoter of *VviLBDIa3* was one of the promoters of LBD genes with AuxRR-core motif involved in auxin responsiveness. The Arabidopsis ortholog of *VviLBDIa3*, *LBD18/ASL20*, together with *LBD16* and *LBD*29, are key regulators of lateral root initiation/formation as direct targets of AUXIN RESPONSE FACTORs^7,19,23^. Arabidopsis *LBD16, 17, 18* and *29* were also found to have an important role in *in vitro* callus formation induced by auxin^9^. Furthermore, both Arabidopsis and banana *LBD* genes were shown to directly regulate expression of *EXPANSIN* genes, encoding cell wall-loosening factors^61–64^ that are also modulated during grape ripening^65^.

Other LBD genes such as *VviLBDIi1* were less expressed during grape ripening. In fact, this gene as well as *VviLBD1c3* were identified as negative biomarkers of ripening stage in three Portuguese cultivars^59^. Additionally, *VviLBDIi1* possesses a *cis*-acting element involved in ABA responsiveness, a growth regulator that increases during ripening^55^, suggesting that this *LBD* gene might be negatively regulated by ABA.

Brassinosteroids are steroidal plant hormones that have been proposed as ripening promoters in non-climacteric fruits, in particular grape berries^55^. The Arabidopsis *LOB* gene negatively regulates the accumulation of brassinosteroids in organ boundaries^66^. *VviLBDIc1* is an ortholog of *LOB* and seems to be down-regulated during grape ripening as suggested here and in previous studies^59^, and possibly interacts with brassinosteroids. However, it should be noted that at pea-size stage of berry development *VviLBDIc1* expression seems to be very low compared to the following ripening stages highlighting the importance of conducting detailed temporal studies of gene expression.

Altogether *VviLBD1d6, VviLBDIa3* and *VviLBD1c3* are robust candidates to participate in the regulation of the onset of the grape ripening program.

### Expression of LBD genes upon abiotic and biotic stresses

In grapevine, some class I genes showed differential expression under abiotic stress conditions. Particularly, *VviLBDIi2* is up-regulated under salt, cold and water deficit conditions, *VviLBDId5* is up-regulated after *in vitro* ABA treatment, and *VviLBDId1*, down-regulated after 48 h of high light exposure. Interestingly, *VviLBDIi2* presents in his promoter a MYB binding site involved in drought-inducibility, *VviLBDId5* a *cis-*acting element involved in the abscisic acid responsiveness and *VviLBDId1* many elements involved in light responsiveness, suggesting the involvement of LBDs in abiotic stress response.

Few studies focused on the role of *LBD* genes in abiotic stress. However, in *Medicago truncatula*, *LBD1* gene was reported to have an important role in root architecture under salt stress^67^. Additionally, MtHB1, an ABA and salinity responsive transcription factor, was found to directly recognize a specific *cis*-acting element in the *MtLBD1* promoter^68^. By contrast, several *Sorghum bicolor LBD* genes where highly induced under salt and drought stress conditions, suggesting a role in abiotic stress response^50^, whereas, in banana fruit, *MaLBD5* expression was induced by cold and methyl jasmonate treatment^69^.

The majority of *VviLBDs* seem to participate in the early response towards *Botrytis* attack as previously mentioned. However, *VviLBDId6* and *VviLBDIIa1* were strongly up-regulated after *Botrytis* infection both at EL33 (green berries) and EL35 (*véraison*)*. VviLBDIIa1* was also found co-expressed with six wound-induced genes as previously referred (Table 2, Supplementary Table S5). Interestingly, the promoter of this gene presents several *cis*-elements related to abiotic stress and methyljasmonate responses. Jasmonates were previously proposed to be involved in grape response to *Botrytis* infection^70^. Expression profiles of several Class II *AtLBD* genes revealed induction by pathogens, including necrotrophic fungal pathogens *Alternaria brassicicola* and *B. cinerea*, root pathogen *Phytophthora parasitica* (oomycete) and the root-knot nematode *Meloidogyne incognita*, suggesting a role in plant defence mechanisms^44^. *VviLBDIIa3* seems to be involved in GA regulation of fruit set and fruit ripening but this gene was also up-regulated upon *Botrytis* infection with higher expression at *véraison* stage. Although gibberellins are mainly associated with plant growth and development, they have been recently related to response to pathogen attack^71^. Still, further studies are required to elucidate the role of GA in plant defence that remains very complex and unclear. Genes coding for gibberellin 20 oxidase were up-regulated after *B. cinerea* infection^70^, suggesting activation of GA metabolism in defence response possibly with the involvement of *VviLBDIIa3*.

The involvement of the *LBD* genes in stress response has been poorly studied so far though some genes have been shown to play a role in disease susceptibility^2^. *AtLBD20* was the first *LBD* gene associated with disease susceptibility^10^. In grapevine, *AtLBD20* homolog/ortholog did not show a relevant expression level in berries upon *B. cinerea* fungal infection, however *VviLBDIa3*, belonging to the same clade, was up-regulated after long exposure to *B. cinerea* inoculation. In addition, *VviLBDIa3* gene was found to be co-expressed with several genes including a NBS-LRR gene (VIT_17s0000g09030) related to defence and a pectinesterase gene (VIT_15s0048g00500), involved in cell wall modification processes. Nucleotide-binding site (NBS) leucine-rich repeats (LRR) proteins are involved in the recognition of pathogen effectors with virulence functions^72^.

Besides *VviLBDIa3* and *VviLBDIIa3, VviLBDIf5* was up-regulated upon *Botrytis* infection at *véraison* stage. Although no ortholog could be found for this gene, it belongs to the clade If that comprises the *AtLBD1* and *AtLBD11*. The closest *Citrus sinensis* homolog of these Arabidopsis genes, *CsLOB1*, was found to function as disease susceptible gene in citrus bacterial canker, a disease caused by multiple *Xanthomonas* species^45^. Moreover, *CsLOB2* and *CsLOB3*, belonging to the same clade as *CsLOB1*, were found to have a similar role as *CsLOB1* in citrus bacterial canker^73^. Another putative disease susceptible gene might be *VviLBDIi2* which was down-regulated in partially and completely resistant plants derived from *Muscadinia rotundifolia* when inoculated with *Plasmopara viticola*. Interestingly, *VviLBDIi2* presented in its promoter *cis*-acting elements involved in salicylic acid responsiveness, a hormone known to be involved in response to biotrophic pathogens^70^.

The involvement of grapevine LBD genes in response to biotic stress was also noticed for Bois noir disease. *VviLBDIc3* showed down- regulation in inflorescences presenting Bois noir disease, in grape berries after long exposure to *B. cinerea*, cold and ABA treatment, which could suggest that some LBD genes may be simultaneously modulated by abiotic and biotic stress conditions.

## Conclusions

LOB domain (LBD) transcription factors families have been characterized in several plant species and shown to participate in the regulation of developmental programs and stress responses. Nevertheless, the role of LBDs in fruit ripening has been poorly documented. Modulation of *LBD* genes expression during grape berry development and ripening indicates that these processes may be under regulation of LBD transcription factors. In addition, several grapevine *LBD* genes bared *cis-*elements in their 5′ regulatory region associated with defence and hormonal regulation which together with expression and co-expression analyses supports their involvement in the abiotic and biotic stress response mechanisms. Candidate genes were identified that exhibit broad response to stress (e.g. *VviLBDIc3)* or could be involved in grape ripening and grape defence (e.g.*VviLBDId6)*. Altogether this data may be used for functional characterization of genes and ulterior improvement of fruit quality traits and resilience to abiotic and biotic stresses.

## Methods

### Identification of *LBD* genes

Genes previously identified as encoding LOB domain proteins in Grimplet *et al*.^30^ were blasted (blastp and tblastn) against the grapevine genome 12Xv.2 (https://urgi.versailles.inra.fr/Species/Vitis/Data-Sequences/Genome-sequences), the non-redundant list of genes in Grimplet *et al*.^30^, the NCBI refseq (both remapped on the 12Xv2 of the genome assembly) and the COST annotation gene set available at the ORCAE website (http://bioinformatics.psb.ugent.be/orcae/). Results from different analyses were manually cross-checked to identify new potential loci corresponding to *LBD* genes in the grapevine genome. The UGene software^74^ was used to design the gene models on the grapevine genome and test their structure.

### Gene structure analysis

The potential coding DNA sequences (CDS) were blasted (blastx) against the NCBI public database to compare the structures with other known *LBD* genes in other species and the NCBI Refseq predictions of the grapevine genes. When discrepancies were observed, gene models were corrected using the UGene software. Loci bearing non-functional genes were eliminated from the list. A GFF file with the *LBD* genes was designed, uploaded into the IGV software and the RNAseq data available on flowers in the laboratory were used to double-check the exon structure of the genes.

### Promoter analysis

Promoter *cis*-acting regulatory elements within 1.5 kb of the upstream sequence from the ATG initial codon of each grapevine *LBD* gene were analyzed with PlantCARE software^75^ (http://bioinformatics.psb.ugent.be/webtools/plantcare/html/). Analysis of transcription factor binding sites (TFBSs) of the 3 kb upstream sequence region of the initial codon was also performed using the Plant Promoter Analysis Navigator (PlantPAN) software^76^ (http://plantpan2.itps.ncku.edu.tw/).

### Enrichment of *cis*-regulatory elements

Motif analysis of known and *de novo* motifs was performed using Homer v4.9^77^ (http://homer.ucsd.edu/homer/motif/). With this end, grapevine promoter sequences (2.5 kb upstream of the coding sequence) of *LBD* genes were retrieved from Regulatory Sequence Analysis Tools (RSAT, http://floresta.eead.csic.es/rsat/). Additionally, in order to prevent overlapping between neighbouring genes, noorf option was performed.

### Sequence alignment and phylogenetic analysis

Sequence information on previously reported LOB domain proteins of *A. thaliana* was retrieved from the Arabidopsis Information Resource (https://www.arabidopsis.org/). Evolutionary analyses were conducted in MEGA6^41^. Multiple sequence alignment was inferred using MUSCLE^78^. The evolutionary history was inferred by using the Maximum Likelihood method based on the JTT matrix-based model^79^. The bootstrap consensus tree inferred from 100 replicates was taken to represent the evolutionary history of the taxa analyzed^80^. Branches corresponding to partitions reproduced in less than 30% of bootstrap replicates were collapsed. Initial trees for the heuristic search were obtained automatically by applying Neighbor-Join and BioNJ algorithms to a matrix of pairwise distances estimated using a JTT model, and then selecting the topology with superior log likelihood value. The coding data was translated assuming a standard genetic code table. All positions with less than 95% site coverage were eliminated. The genes were named according to Grimplet and co-workers^32^ based on the distance homology with Arabidopsis genes.

The alignment file between Arabidopsis and grapevine sequences was uploaded to the Jalview and UGene software for manual adjustment of the alignment and manual motif editing. Motifs were flagged and labelled for the grapevine genes; additional motifs of high homology were also identified (at least 50% homology within the members of the subfamily on at least 10 amino acids) among grapevine sequences.

### Expression analysis

Expression data were retrieved from 3 different microarray platforms (Affymetrix Genchip (16k probesets) GrapeGen (21k probesets), Vitis Nimblegen array (29k probesets) and from our in-house RNAseq projects. Data normalization was performed on all the array of each platform (RMA normalization). After retrieving the values for the probesets corresponding to each gene, the values for the 3 or 4 replicates of the same condition were averaged to obtain a total of up to 256 conditions (organ, cultivar, treatment, platform) for the genes present in all platform. Based on expression data of the grapevine gene expression atlas^37^, a plant ontology ID was attributed to each gene if expression intensity in a tissue was above a defined threshold of absolute log_2_ value of 8 or absolute value of 256. The same data were used for the co-expression analysis with the whole set of genes available on the Nimblegen platform. Hierarchical clustering with Pearson correlation as metric and average linkage cluster method was performed. Genes considered as having the same profile should present a distance threshold between each other lower than of 0.2.

For further evaluation of gene expression samples corresponding to several stages of grapevine development and ripening and several abiotic and biotic stress conditions were used^37,39,65,70,81–96^. Heat maps were performed with the ComplexHeatmap R package (https://github.com/jokergoo/ComplexHeatmap).

### Sequence comparison among diverse plant species

We performed a sequence comparison using the LBD genes from 33 plant species (*Arabidopsis thaliana, Brassica rapa, Carica papaya, Theobroma cacao, Gossypium raimondii, Eucalyptus grandis, Citrus clementina, Manihot esculenta, Ricinus communis, Populus trichocarpa, Linum usitatissimum, Malus domestica, Pyrus bretschneideri, Prunus persica, Fragaria vesca, Cicer arietinum, Glycine max, Medicago truncatula, Citrullus lanatus, Cucumis sativus, Solanum lycopersicum, Utricularia gibba, Nelumbo nucifera, Hordeum vulgare, Triticum aestivum, Oryza sativa subsp. indica, Phyllostachys heterocycla, Sorghum bicolor, Zea mays, Musa acuminata, Phoenix dactylifera, Picea abies*) retrieved at http://planttfdb.cbi.pku.edu.cn. We identified orthologous genes in genomes from the thirty-three species following what was performed in Jaillon *et al*.^28^. Each pair of predicted gene sets was aligned with the BLASTp algorithm, and alignments with an e-value lower than 1e^−20^ and sequence homology higher than 40% were retained. If a comparison is above that value, the two genes were considered homologs. Two genes, A from Vitis genome (GV) and B from a given species genome (GX), were considered orthologs one-to-one if B was the best match for gene A in GX and A was the best match for B in GV. A phylogenetic tree was constructed with the LBD genes from these species with the same parameters as before.

A Ka/Ks analysis was performed using the Ka/Ks calculation tool (http://services.cbu.uib.no/tools/kaks) on all the orthologs detected in the species for each grapevine gene with the default parameters.

### Data availability statement

All the data published in this article will be available for scientific community.

## Electronic supplementary material

Supplementary Figures

Supplementary Table S1

Supplementary Table S2

Supplementary Table S3

Supplementary Table S4

Supplementary Table S5
