# Supplementary material for: The LATERAL ORGAN BOUNDARIES Domain gene family in grapevine: genome-wide characterization and expression analyses during developmental processes and stress responses
Source: Sci Rep. 2017 Nov 21;7:15968. doi: 10.1038/s41598-017-16240-5 (PMC5698300; doi:10.1038/s41598-017-16240-5)

The *LATERAL ORGAN BOUNDARIES Domain* gene family in grapevine: genome-wide characterization and expression analyses during developmental processes and stress responses

Jérôme Grimplet[1+], *Diana Pimentel*[2+], *Patricia Agudelo-Romero*[2#], *Jose Miguel Martinez-Zapater*[1], *Ana Margarida Fortes*[2 *]

[1]Instituto de Ciencias de la Vid y del Vino (CSIC-Universidad de La Rioja-Gobierno de La Rioja), 26006 Logroño, Spain; [2]Universidade de Lisboa, Faculdade de Ciências de Lisboa, BioISI, Campo Grande, 1749-016 Lisboa, Portugal;

[+] co-authorship

[*]Correspondence:

Ana Margarida Fortes, Universidade de Lisboa, Faculdade de Ciências de Lisboa, BioISI, Campo Grande 1749-016 Lisboa, Portugal. Telephone number: +351 21 7500382; Fax number: +351 217500048. E-mail: amfortes@fc.ul.pt

[#]Present address: The UWA Institute of Agriculture and the ARC Centre of Excellence in Plant Energy Biology, The University of Western Australia, M316 Perth, 6009, Australia

Additional emails: JG (jerome.grimplet@icvv.es), DP (dianalpoim@gmail.com), PA-R (agudeloromero@hotmail.com), JMM-Z (zapater@icvv.es)

SUPPLEMENTARY INFO

Supplementary Datasets

Supplementary Table S1. Complete annotation of the grapevine *LBD* genes and ka/ks analysis.

Supplementary Table S2. Complete *cis*-regulatory elements analysis of *VviLBD* genes promoters with PlanCARE software.

Supplementary Table S3. Complete analysis of transcription factor binding sites analysis in *VviLBD* genes promoters with PlantPAN software.

Supplementary Table S4. Motifs enrichment data. Identification of known and *de novo* motifs using Homer software. List of LOB genes containing the *de novo* motif (GGTTGAATACAC); the scan was performed allowing up to two substitutions.

Supplementary Table S5. List of co-expressed genes with *LBD* genes.

Supplementary Figures

Supplemental Figure S1. Exon-intron structure of the 50 grapevine *VviLBD* genes. Exons are represented by yellow boxes and introns by black lines. The majority of *VviLBD* genes presented two exons (37 genes). Gene structures of the *VviLBD* genes were generated with the GSDS 2.0 [96](http://gsds.cbi.pku.edu.cn/).

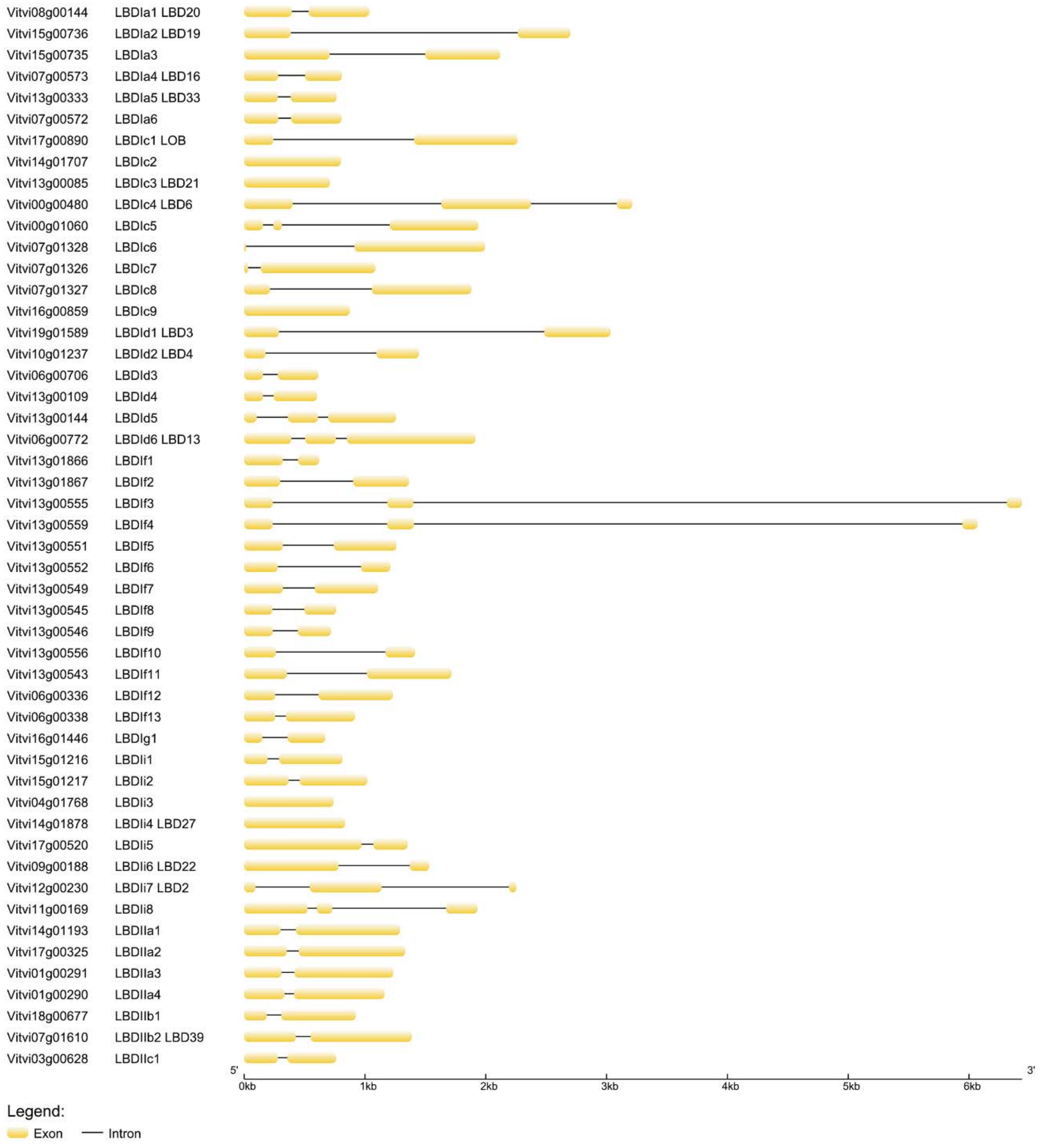

Supplementary Figure 2. Grapevine LBD proteins alignment.

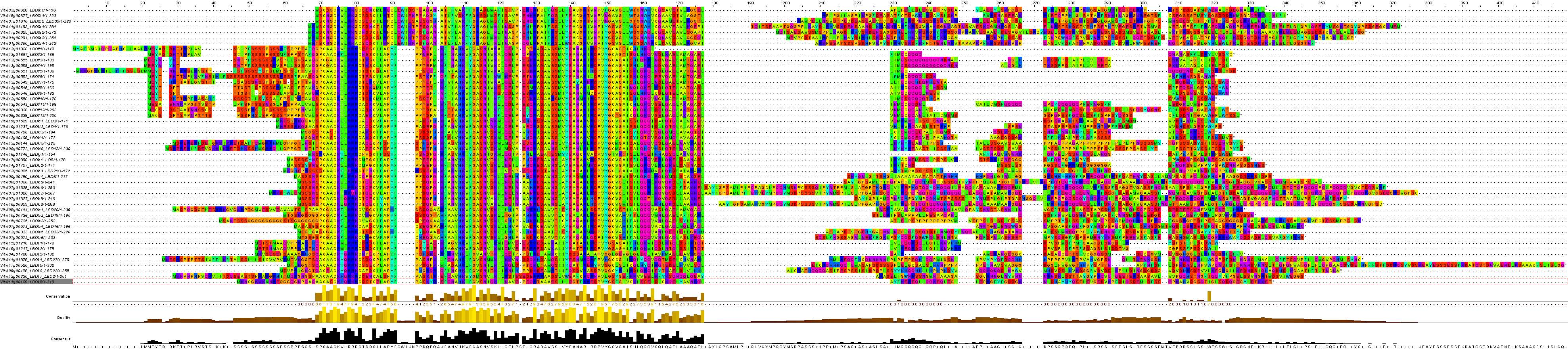
Conservation
Quality
Consensus

Supplementary Figure 3. Molecular phylogenetic analysis by Maximum Likelihood method between grapevine and 33 plant species.

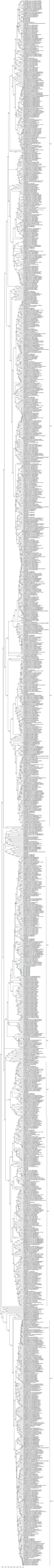

Supplementary Figure S4. Distribution of *cis*-regulatory elements in the *VviLBD* promoter regions. Bar graph depicts the total number of genes for each *cis*-element motif. Several regulatory motifs were identified as associated with light regulation, defense and stress responses, and hormonal regulation.

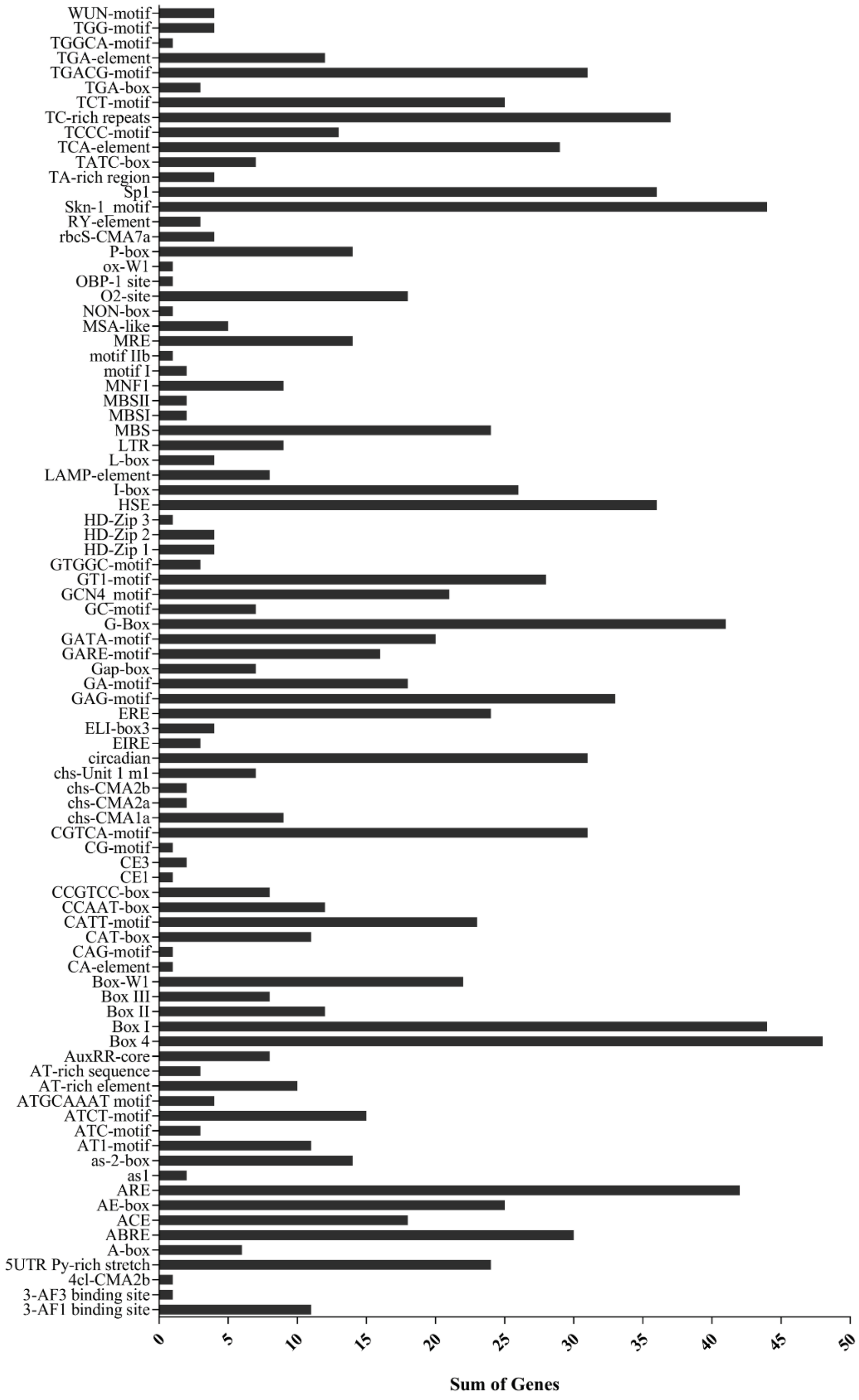

Supplementary Figure S5. Distribution of transcription factor binding sites in the *VviLBD* promoter regions. Bar graph depicts the total number of genes for each transcription factor binding sites motifs. Several transcription factor binding sites were identified as associated with developmental processes and with biotic and abiotic stress responses.

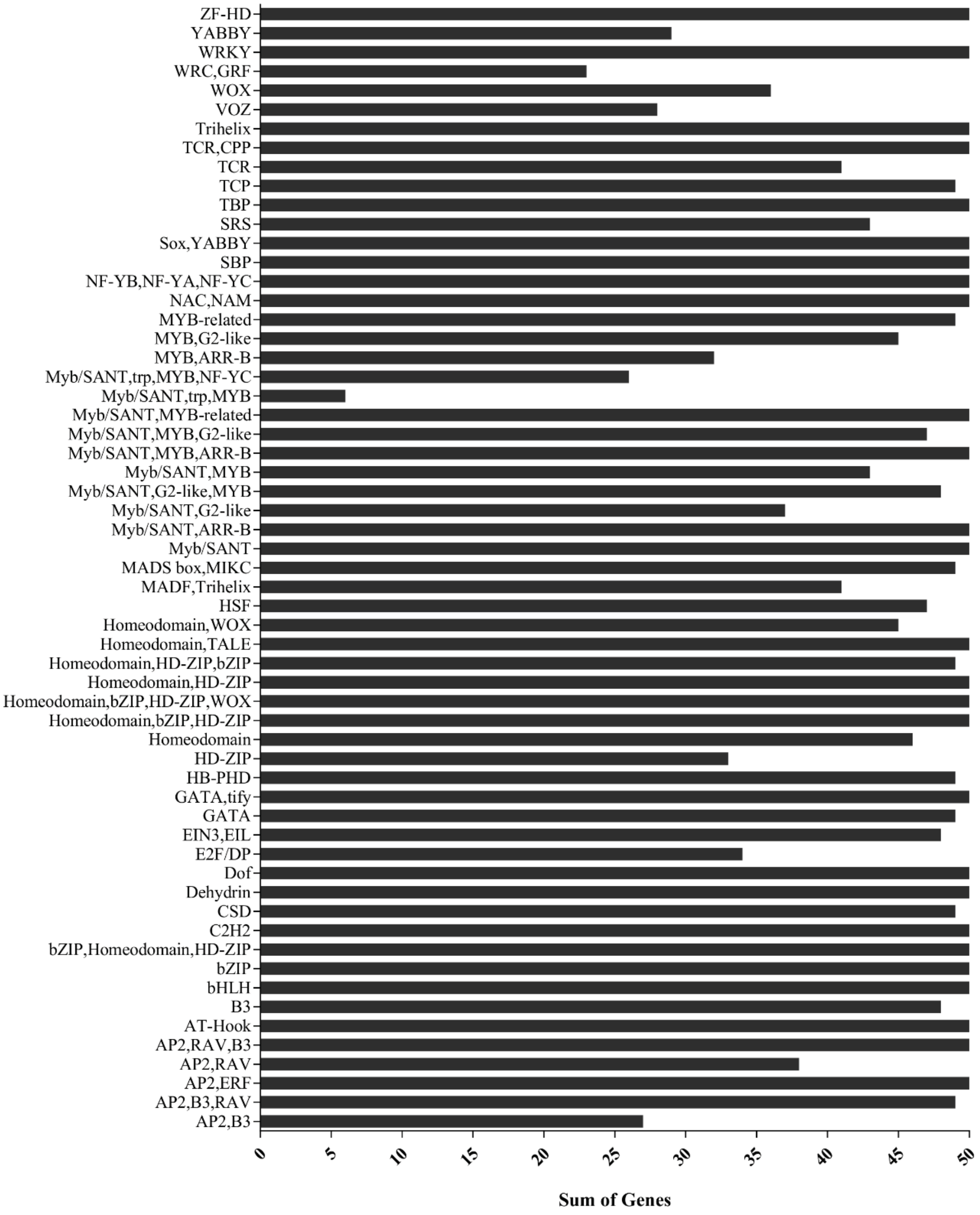

Supplement: Supplementary file 1 — Supplementary Figures [file 41598_2017_16240_MOESM1_ESM.pdf]
